# Spatiotemporal diversity and community structure of cyanobacteria and associated bacteria in the large shallow subtropical Lake Okeechobee (Florida, United States)

**DOI:** 10.3389/fmicb.2023.1219261

**Published:** 2023-08-30

**Authors:** Forrest W. Lefler, Maximiliano Barbosa, Paul V. Zimba, Ashley R. Smyth, David E. Berthold, H. Dail Laughinghouse

**Affiliations:** ^1^Agronomy Department, Fort Lauderdale Research and Education Center, University of Florida—IFAS, Davie, FL, United States; ^2^Rice Rivers Center, Virginia Commonwealth University, Charles City, VA, United States; ^3^Soil, Water and Ecosystem Sciences Department, Tropical Research and Education Center, University of Florida—IFAS, Homestead, FL, United States

**Keywords:** harmful algal blooms, *Microcystis*, *Dolichospermum*, eutrophication, picocyanobacteria, metabarcoding, microbiome

## Abstract

Lake Okeechobee is a large eutrophic, shallow, subtropical lake in south Florida, United States. Due to decades of nutrient loading and phosphorus rich sediments, the lake is eutrophic and frequently experiences cyanobacterial harmful algal blooms (cyanoHABs). In the past, surveys of the phytoplankton community structure in the lake have been conducted by morphological studies, whereas molecular based studies have been seldom employed. With increased frequency of cyanoHABs in Lake Okeechobee (e.g., 2016 and 2018 *Microcystis*-dominated blooms), it is imperative to determine the diversity of cyanobacterial taxa that exist within the lake and the limnological parameters that drive bloom-forming genera. A spatiotemporal study of the lake was conducted over the course of 1 year to characterize the (cyano)bacterial community structure, using 16S rRNA metabarcoding, with coincident collection of limnological parameters (e.g., nutrients, water temperature, major ions), and cyanotoxins. The objectives of this study were to elucidate spatiotemporal trends of community structure, identify drivers of community structure, and examine cyanobacteria-bacterial relationships within the lake. Results indicated that cyanobacterial communities within the lake were significantly different between the wet and dry season, but not between periods of nitrogen limitation and co-nutrient limitation. Throughout the year, the lake was primarily dominated by the picocyanobacterium *Cyanobium*. The bloom-forming genera *Cuspidothrix*, *Dolichospermum*, *Microcystis*, and *Raphidiopsis* were highly abundant throughout the lake and had disparate nutrient requirements and niches within the lake. Anatoxin-a, microcystins, and nodularins were detected throughout the lake across both seasons. There were no correlated (cyano)bacteria shared between the common bloom-forming cyanobacteria *Dolichospermum*, *Microcystis*, and *Raphidiopsis*. This study is the first of its kind to use molecular based methods to assess the cyanobacterial community structure within the lake. These data greatly improve our understanding of the cyanobacterial community structure within the lake and the physiochemical parameters which may drive the bloom-forming taxa within Lake Okeechobee.

## 1. Introduction

Shallow lakes are sensitive to anthropogenic influences (Scheffer, 2004) and cyanobacteria can often dominate the phytoplankton community of eutrophic shallow lakes, especially in warm climates (Reynolds, 1987; Bonilla et al., 2023). Lake Okeechobee is a large shallow subtropical lake in peninsular Florida (United States), that has been undergoing anthropogenic induced eutrophication since the 1970’s (Canfield et al., 2021). The accumulation of nutrients has resulted in an increase in cyanobacterial dominance of the phytoplankton community leading to cyanobacterial harmful algal blooms (cyanoHABs). Lake Okeechobee has a humid subtropical climate and experiences wet (May through November) and dry (November through May) seasons. The lake has a mean depth of ~2.7 m (Havens et al., 1994) and a large drainage basin (12,000 km^2^), which begins in Orlando running through the Kissimmee Chain of Lakes via the Kissimmee River, flowing south before emptying into the northern region of the lake. This inflow accounts for the majority of the input, with lesser inputs from Lake Istokpoga and Fisheating Creek (Zhang Y. et al., 2020; Canfield et al., 2021). Lake outflow is controlled by the United States Army Corps of Engineers through three main tributaries: south through the Everglades Agricultural Area and ultimately into Florida Bay, west through the Caloosahatchee River into the Gulf of Mexico, and east via the St. Lucie Canal into the St. Lucie Estuary. Land use in the drainage basin is predominantly agricultural (~46%), but urban and suburban areas also exist contributing to the increased nutrient inputs into the lake (Zhang et al., 2011).

Prior to 1974, records show that the phytoplankton community of Lake Okeechobee consisted of <30% cyanobacteria (Marshall, 1977); however, by the 1980’s, the community structure had shifted to a cyanobacteria dominated community (>60%) due to increased eutrophication (Cichra et al., 1995; Havens et al., 1998). This shift to a cyanobacterial dominated community corresponded with an increase in cyanoHABs within Lake Okeechobee. In the past (1970’s–1980’s), cyanoHABs were dominated by diazotrophic cyanobacteria (i.e., *Aphanizomenon*, *Dolichospermum*, *Raphidiopsis*; Joyner, 1974; Marshall, 1977; Jones, 1987), whereas current blooms are often dominated by the non-diazotrophic species *Microcystis aeruginosa* (Kützing) Kützing; although *M. aeruginosa* blooms have been reported as early as 1973 (Davis and Marshall, 1975). Despite this, *Dolichospermum* and *Raphidiopsis* dominated blooms still occur within the lake, though blooms composed of these genera are less frequent and intense than those composed of *Microcystis*. These three notorious genera are known to form cyanoHABs globally and have disparate nutrient requirements where *Dolichospermum* is known to proliferate in low nitrogen conditions, whereas *Microcystis* prefers high nitrogen, low phosphorus concentrations (Werner and Laughinghouse, 2009; Li and Li, 2012; Chia et al., 2018; Werner et al., 2020). These bloom-forming genera can also produce several cyanotoxins (e.g., anatoxins, cylindrospermopsins, microcystins), resulting in deleterious effects to aquatic systems and human health (O’Neil et al., 2012; Huang and Zimba, 2019).

Environmental drivers of cyanoHABs and bloom-forming genera have been studied in detail (e.g., O’Neil et al., 2012; Paerl et al., 2016). Much of the historical focus was on the role of phosphorus (P) on cyanobacteria productivity, known as the P-only paradigm, although there was a recent shift to focus on the role of both nitrogen (N) and P in bloom proliferation (Paerl et al., 2016). External nutrient loading into Lake Okeechobee, primarily as P, has decreased water quality and total phosphorus (TP) concentrations have nearly doubled since the 1970’s, while total nitrogen (TN) concentrations have remained relatively stable (James and Pollman, 2011). Additionally, much of the P in the lake is legacy phosphorus bound to sediment which, when resuspended, can further increase P concentrations (i.e., internal loading; Moore et al., 1998; Fisher et al., 2005). Because of this increased P loading, primary productivity within Lake Okeechobee has been considered N-limited (Havens, 1995; Havens et al., 2003; Kramer et al., 2018), and periods of increased N loading into the lake have increased cyanoHABs (Havens et al., 2016; Lapointe et al., 2017; Kramer et al., 2018).

In fresh waters, the bacterioplankton community (including cyanobacteria) play critical roles in biogeochemical cycles (e.g., carbon, N, P; Falkowski et al., 2008). Bacteria can form symbiotic relationships with cyanobacteria, either as epibionts on colonies, known as the phycosphere (Bell and Mitchell, 1972) or as co-existing, free living, taxa (Morris et al., 2011). The associated bacteria are capable of filling in missing genomic functions (e.g., vitamin synthesis, nitrogen cycling; Morris et al., 2011; Garcia et al., 2015) and form mutualistic relationships with cyanobacteria (Cook et al., 2020). Thus, associated bacteria have the potential to increase the fitness of cyanobacteria, such as intensifying their growth rate (Jackrel et al., 2021). Despite their close relationships, the role of bacteria in cyanoHABs and relationships with bloom-forming cyanobacteria are often overlooked (Pound et al., 2021). Furthermore, the majority of the focus on bacterial-cyanobacterial interactions have centered on the phycosphere (i.e., epibionts or particle-associated) bacteria, with less focus on the bacterioplankton (i.e., free-living; Louati et al., 2023).

High-throughput sequencing (HTS) facilitates insights into microbial community via sequencing taxonomically informative regions (i.e., metabarcoding), such as the 16S rRNA, or whole genome sequencing (metagenomics). Metabarcoding is used extensively for bacterial communities, including the characterization of the cyanobacterial community (e.g., Pessi et al., 2016; Huang et al., 2020; Khomutovska et al., 2020) as these methods provide valuable information on the cyanobacterial community structure and provide increased taxonomic resolution compared to traditional morphological evaluations alone (MacKeigan et al., 2022).

Extensive research has investigated the global/general drivers of cyanoHABs, with much of the focus on *Microcystis* and *Dolichospermum* and intergeneric competition (e.g., O’Neil et al., 2012; Paerl and Otten, 2013; Paerl and Otten, 2016; Almanza et al., 2019; Shan et al., 2019). Within Lake Okeechobee, previous research has studied how various limnological parameters affect shifts within the cyanobacterial community (Havens et al., 1998; Ma et al., 2022), the diversity of phytoplankton including bloom forming genera (Cichra et al., 1995; Ma et al., 2022), and the drivers of increased algal abundance (as chlorophyll; Havens et al., 1994; Xu et al., 2022); However, the specific drivers of bloom forming cyanobacterial genera within Lake Okeechobee remain unexplored. Considering the dominance of cyanobacteria within Lake Okeechobee and the increased frequency and intensity of cyanoHABs (e.g., 2016 and 2018 *Microcystis* blooms), it is imperative to characterize the cyanobacterial community to identify spatial and temporal trends of common bloom-forming genera, specifically *Dolichospermum*, *Microcystis* and *Raphidiopsis*, and elucidate their respective environmental drivers within this system.

Over the course of 1 year, six sites within Lake Okeechobee were sampled for 16S rRNA metabarcoding analysis and limnological parameters to characterize the cyanobacterial and associated bacterial community. Our objectives were to (1) characterize (temporally and spatially) the cyanobacterial community structure within Lake Okeechobee, (2) elucidate the limnological parameters that potentially drive cyanobacterial abundance, and (3) examine cyanobacterial-bacterial relationships. To our knowledge, a spatiotemporal assessment using molecular methods has yet to be conducted on Lake Okeechobee and this study is the first of its kind.

## 2. Materials and methods

### 2.1. Study area and limnological parameters

**Figure 1:**
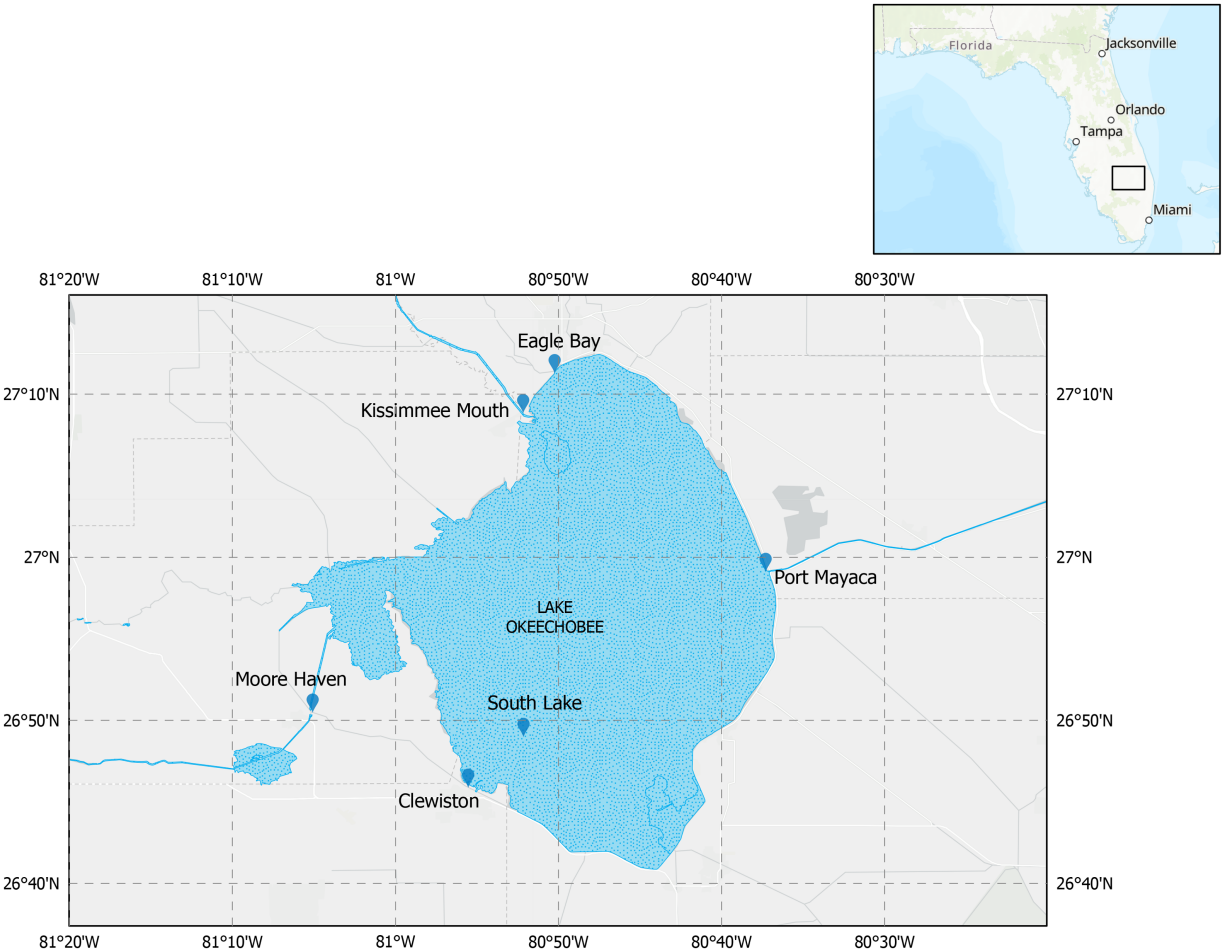
Map of Lake Okeechobee with the sampling locations.

Sampling on Lake Okeechobee occurred 10 times over the course of 1 year (August 2019–September 2020) at approximately five-week intervals at six locations within the lake, Figure 1. Surface water samples (<0.5 m depth) were collected using acid washed and sterile 1 L Nalgene bottles for environmental DNA extractions and stored on ice until processing. Additional water samples were collected for nutrient analyses (i.e., nitrate, nitrite, ammonium, orthophosphate, and total reactive phosphorus) and major ion analysis (i.e., boron, copper, calcium, potassium, sodium, iron, cobalt, magnesium, manganese, aluminum, and zinc). For orthophosphate and major ion analysis, samples were filtered through a 0.45 μm glass filter (MilliporeSigma, Burlington, MA, United States) in the field and the latter acidified with nitric acid. All samples were kept on ice until processing. Water quality measurements (i.e., dissolved oxygen, water temperature, pH, salinity, conductivity, turbidity, chlorophyll-*a* abundance, and phycocyanin abundance) were gathered using a YSI EXO3 (Xylem Inc., OH, United States) multiparameter sonde on site. Secchi depth was measured using a Secchi disk and used to estimate water transparency and to calculate photic depth (Zeu). Samples for cyanotoxin analysis were collected in 250 mL HDPE amber bottles. A total of 57 samples were collected during this study.

Immediately upon arrival in the laboratory, water samples for eDNA were filtered through 0.7 μm Whatman glass filters (GF/F MilliporeSigma, Burlington, MA, United States) until clogging and stored at −80°C. Water samples for nutrient composition analysis were frozen and stored, except orthophosphate, which was kept at 4°C until processing. Total reactive phosphorus (TRP; i.e., unfiltered), nitrate, nitrite, and ammonium were analyzed using Standard Methods 4500 (APHA, 2017) on a Seal AutoAnalyzer (Seal AA500; Seal Analytical, WI, United States). Trace elements were quantified using an Avio 200 ICP-OES (Inductively Coupled Plasma Optical Emission Spectrometer) following Standard Method 3,120 (Baird and Bridgewater, 2017). Additional water chemistry parameters were obtained from the South Florida Water Management Districts DBHydro database.^1^

### 2.2. Cyanotoxin analysis

Mass spectrometry multiple reaction monitoring (MS-MRM) was used to analyze samples from all collection sites for multiple microcystin (MC) congeners as well as nodularin (NOD), saxitoxin (STX), and cylindrospermopsin (CYN). Water samples were frozen and thawed three times, then concentrated using C18 sorbent (Strata-X, Phenomenex Corporation, Torrance, CA, United States, 60 mg sorbent, 3 mL syringe volume). After elution, samples were placed into autosampler vials for high performance liquid chromatography tandem mass spectrometry (HPLC-MS/MS) analysis on an Agilent 1200 series HPLC in-line with an Agilent 6410b triple quadrupole mass spectrometer (Agilent, Santa Clara, CA, United States) fitted with an electrospray ionization source. The autosampler was maintained at 8°C and injected 40 μL of sample. The analytes were passed through a column shield prefilter (MAC-MOD Analytical, Inc., Chadds Ford, PA, United States) and loaded onto a Luna C18(2), 3-μm particle size, 150 × 3 mm column (Phenomenex Corporation, Torrance, CA, United States) heated to 35°C with 100% mobile phase A (90% water, 10% acetonitrile, 0.1% formic acid) at a flow rate of 0.4 mL min^−1^. Initial conditions were maintained for 2 min, and analytes were eluted over a six-minute gradient from 0% to 90% mobile phase B (100% acetonitrile, 0.1% formic acid) followed by 3 min at 90% mobile phase B, before returning to initial conditions for 3 min. MS/MS analysis used Agilent MassHunter Data Acquisition software (version B.02.01, Agilent, Santa Clara, CA, United States). Samples were run in positive ion mode by MS-MRM and full scan mode (*m*/*z* 100–1,200). Data were analyzed using Agilent MassHunter Qualitative Analysis software (version B.03.01, Agilent, Santa Clara, CA, United States). A standard curve (1/y^2^ weighting) was established for each toxin (except MC-LW, which was quantified using the MC-LR standard curve) by integrating the peak area of the quantifier ion from duplicate standards (6 concentrations ranging from 0 to 10 ng μL^−1^), with a limit of detection of 0.5 ng on the Phenomenex column. Standards were prepared in methanol and analyzed in the same manner as the samples. To measure the amount of each toxin in the samples, the peak area of the quantifier ion was compared to the appropriate standard curve. The limit of detection of each toxin in water is 0.0003–0.0009 μg L^−1^ microcystin (varies based on congener), 0.0005 μg L^−1^ cylindrospermopsin, and 0.0009–0.0013 μg L^−1^ saxitoxin. Standards for toxin analysis included various sources for microcystins including Enzo Life Sciences (Farmingdale, NY, United States), Cayman Chemical (Ann Arbor, MI, United States), Greenwater Laboratories (Palatka, FL, United States), and CCS purification. Pure saxitoxin standards were purchased from Cayman Chemical (Ann Arbor, MI, United States) and additional material was isolated from a toxic strain of *Dolichospermum circinale* (obtained from Dr. Brett Neilan). Cylindrospermopsin standards were obtained from Dr. Brett Neilan.

### 2.3. DNA extraction, amplicon library preparation, and processing

DNA was extracted using a DNeasy Blood and Tissue Kit (Qiagen, Hilden, Germany), modified according to Djurhuus et al. (2017). The V4–V5 hypervariable regions of the 16S rRNA were amplified using 515FY-926R primer pair described in Parada et al. (2016). Samples were amplified in triplicate before pooling. Amplicon libraries were sequenced using paired-end (2 × 250 bp) Illumina Novaseq (Novogene, Beijing, China), sequencing depth varied from 88,722 to 139,935 reads per samples with a mean of 129,647. The V3–V4 variable regions of the 16S rRNA were obtained by using both sets of cyanobacterial specific primer pairs (i.e., CYA359F-781Ra/b) described by Nübel et al. (1997). However, these produced a low number of cyanobacterial ASV’s due to amplification of eukaryotic phytoplankton chloroplast 16S rRNA sequences and were thus excluded from analysis (data not shown).

Amplicon sequences were demultiplexed and assigned to specific sample IDs based on their MIDs at Novogene using an in-house bioinformatic pipeline. DADA2 (Callahan et al., 2016) was used to process raw sequences in R v4.0.0 (R Core Team, 2023). Paired-end reads were filtered, trimmed, and merged. Cleaned and merged reads were dereplicated and subsequently analyzed for detection and removal of potential chimeras using DADA2. Non-chimeric sequences were pooled to define amplicon sequence variants (ASVs) and identical ASVs which only varied in length were collapsed using the “collapseNoMismatch” command in DADA2, ASVs ranged in length from 325 to 393 nt.

Taxonomic assignment of ASVs was based on a naïve Bayesian classifying method (Wang et al., 2007) with CyanoSeq V1.2 (Lefler et al., 2023) and SILVA 138.1 (Quast et al., 2012) as the taxonomic databases. The CyanoSeq database was supplemented with 16S rRNA sequences from unialgal cyanobacterial cultures isolated from Lake Okeechobee and surrounding fresh waters housed in the Berthold Laughinghouse Culture Collection (BLCC) at the University of Florida – IFAS, Fort Lauderdale Research and Education Center (Davie, FL, United States). All non-cyanobacterial ASVs, including chloroplasts, were removed prior to downstream analyses. All archaeal, chloroplast, eukaryotic, and mitochondrial ASVs were removed for network analysis. A maximum likelihood phylogenetic tree of the cyanobacterial ASVs was created using RAxML-NG (Kozlov et al., 2019), by determining the sequence evolutionary model (GTR-I-G4) using ModelTestNG (Darriba et al., 2020). A maximum likelihood phylogenetic tree of the bacterial ASVs was created using IQTree with ultrafast bootstrapping (Minh et al., 2020).

ASV’s which corresponded to the Aphanizomenonaceae and Microcystaceae were extracted, and phylogenetic trees were constructed for each family. Cyanobacterial ASVs that could not be classified to the genus level (except for Prochlorococcaceae) were extracted and placed in the reference tree from CyanoSeq (v1.2) along with their three closest BLAST hits to provide increased resolution. Sequences were added to the alignment using MAFFT (Katoh and Standley, 2013), full length sequences (i.e., >600 bp) were added with—add and—keeplength parameters, while ASVs and short sequences (i.e., <600 bp) were added using—add-fragment and—keeplength parameters. The alignment was trimmed using TrimAl (Capella-Gutiérrez et al., 2009) using -automated1 parameter and the sequence evolutionary model was determined using ModelTestNG (Darriba et al., 2020). The phylogenetic tree was built using RAxML-NG with 1,000 bootstrap replicates (Kozlov et al., 2019).

### 2.4. Statistical and network analyses

Sequence read abundances were filtered using the phyloseq package (McMurdie and Holmes, 2013). ASVs that occurred in less than 10% of samples or occurred less than 100 times across all samples were removed from all downstream analyses, except alpha diversity. The vegan package (Oksanen et al., 2019) was used for statistical analyses, calculation of richness and diversity indices, and generation of ordinations in combination with ggplot2 (Wickham, 2016) in R v4.0.0 (R Core Team, 2023). Alpha diversity was calculated using Faith’s Phylogenetic Distance indices, and Wilcoxon tests were used to compare between groups. Data were not rarefied (McMurdie and Holmes, 2014), prior to analyses, the data were log-transformed (1og10) to avoid biases toward rare species and minimize influence of most abundant groups. Indicator species were determined using the indicspecies package (De Cáceres and Legendre, 2009).

Similarities in cyanobacterial communities among sampling sites and seasons (i.e., wet vs. dry season, nitrogen limitation vs. co-limitation) were explored using the Non-Metric Multidimensional Scaling (NMDS) analysis with generalized Unifrac distances (Chen J. et al., 2012; Chen X. et al., 2012). The “adonis2” function of the vegan package was used to conduct a permutational multivariate analysis of variance (PERMANOVA) on generalized Unifrac distances to test the effect of sampling sites, nutrient limitation, and seasonal impact on cyanobacterial community composition. Partial redundancy analysis (pRDA) was employed using the rda() function in the vegan package to find relationships between significant environmental variables (*p* ≤ 0.05) and *Cuspidothrix*, *Cyanobium*, *Dolichospermum*, *Microcystis, Raphidiopsis*, and *Vulcanococcus* were selected as these were the five most abundant described genera in our data. Environmental variables were standardized based on square root transformation prior to analysis.

Generalized additive models (GAMs) were used to model the relationship between cyanobacterial genera (as rarefied read abundance) and limnological parameters (e.g., water temperature, nutrients, etc.) with sampling sites and outing as random effects. GAMs were conducted in R using the mgcv package (v1.8-42; Wood, 2011) and drawn with gratia (Simpson, 2023) and ggplot2.

Cyanobacterial-bacterial relationships were explored using the Sparse Inverse Covariance Estimation for Ecological Association Inference (SpiecEasi; v1.1.0) package in R (Kurtz et al., 2015) using the top 500 most abundant genera. Networks were visualized in Cytoscape v3.9.1 (Shannon et al., 2003).

## 3. Results

### 3.1. Limnological parameters

Dissolved inorganic nitrogen to dissolved inorganic phosphorus ratio (DIN:DIP) was determined, as was the dissolved inorganic nitrogen (DIN), defined here as the sum of nitrate, nitrite, and ammonia, to soluble reactive phosphate. DIN:DIP ratio ranged from 0.02 to 75, while DIN ranged from 0.05–0.634 mg L^−1^. These data are reported in Supplementary Data S1. Total nitrogen and phosphorus measurements were obtained from the South Florida Water Management Districts DBHydro database; the TN:TP mass ratio ranged from 11 to 71 with a mean of 28. Total nitrogen concentrations ranged from 0.87–3.14 mg L^−1^and total phosphorus concentrations ranged from 0.056–0.392 mg L^−1^. Periods of nutrient limitation were determined by collecting TN and TP data from the sites in closest proximity to our sampling sites and plotting the TN:TP ratio as a time series during our sampling events (Supplementary Figure S1). Only one site indicated phosphorus limitation (TN:TP ≥  23) and was thus excluded from further analyses. Nutrient limitation was based on values provided by Paerl et al. (2016) where N:P ≥ 23 indicates P-limitation, N:P ≤ 9 indicates N-limitation, and 23 > N:P > 9 indicates co-nutrient limitation. Other water quality parameters (e.g., trace elements, conductivity, photic depth) are reported in Supplementary Data S1. Daily mean lake depth was determined from the LZ40 station (lat 26.901815, long −80.789003) and ranged from 3.5–4.7 m.

### 3.2. Cyanobacterial composition

**Figure 2:**
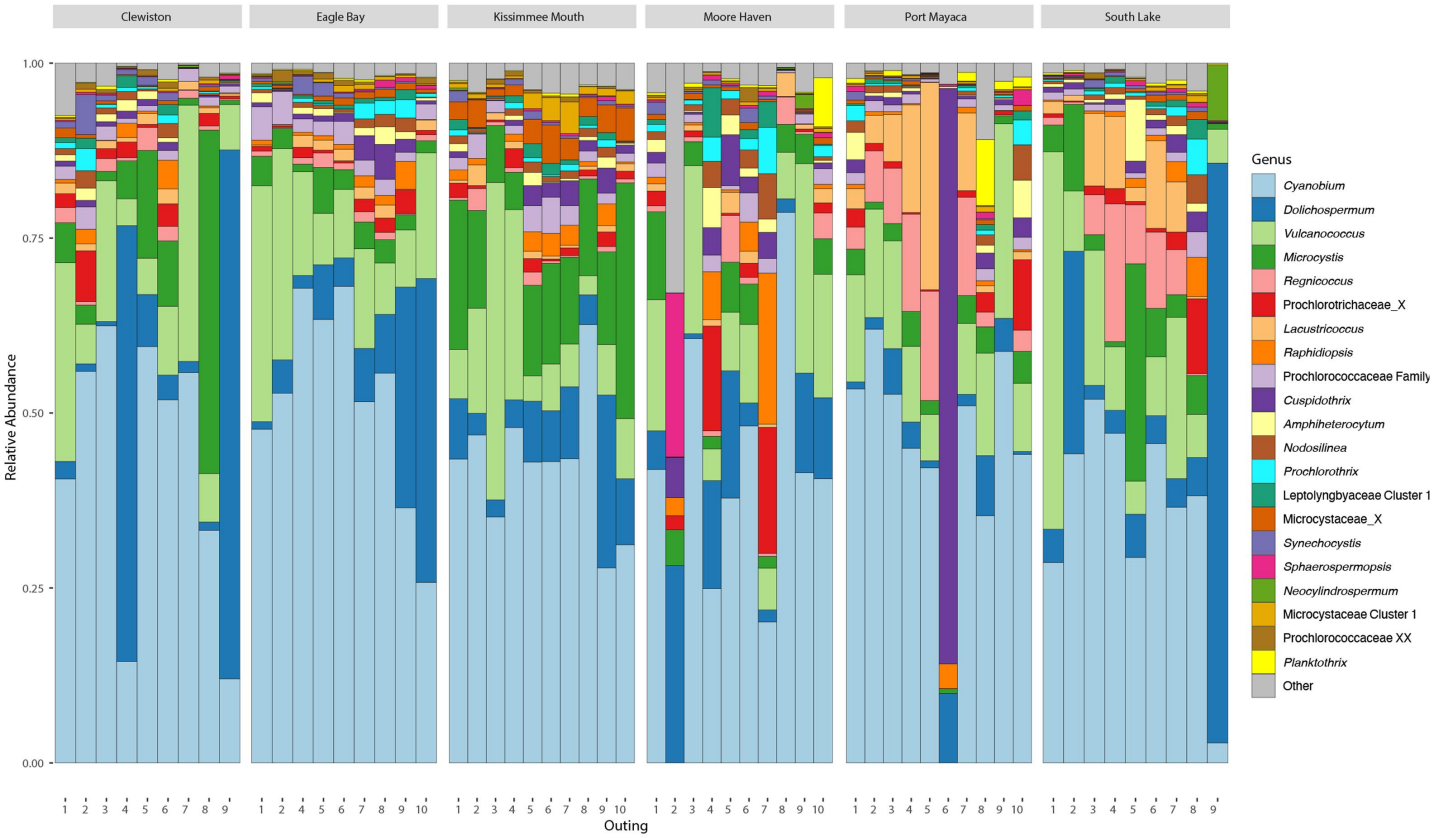
Bar plots showing the relative abundance of most abundant cyanobacterial genera found during the study.

After filtering, there were an average of 67,826 (min = 44,914, max = 84,259, sd = 8,876) reads across samples assigned to 4,048 ASVs, 274 of which were cyanobacteria with an average of 20,168 (min = 2,429, max = 58,317, sd = 11,403) reads. The cyanobacteria, phylum Cyanobacteriota, frequently made up >25% of the total bacterial community (Supplementary Figure S2A). Based on relative read abundance, the Synechococcales, Nostocales, and Chroococcales were the most abundant cyanobacterial orders (Supplementary Figure S2B), with the Prochlorococcaceae, Aphanizomenonaceae, and Microcystaceae as the most abundant families (Supplementary Figure S2C). The most abundant genus within Lake Okeechobee was *Cyanobium*, a member of the Prochlorococcaceae, followed by *Dolichospermum*, and *Microcystis*. The most abundant toxigenic bloom-forming genera were *Dolichospermum* and *Microcystis* (Figure 2), although several other potentially toxic bloom-forming genera were found throughout the lake at lower relative abundances including *Aphanizomenon*, *Cuspidothrix*, *Raphidiopsis*, and *Sphaerospermopsis*.

Due to the high abundance and bloom potential of the Aphanizomenonaceae and Microcystaceae, phylogenetic inferences of these ASVs were conducted to confirm taxonomic assignment (Supplementary Figures S3, S4). All ASVs assigned to a genus were found to be monophyletic with their respective genus. ASV1987 was assigned as “Aphanizomenonaceae” but phylogenetic inferences revealed this belonged to *Amphiheterocytum* and was manually reassigned (Supplementary Figure S3). ASV176 was only assigned to the rank “Aphanizomenonaceae” and formed a well-supported clade away from known genera within the Aphanizomenonaceae, thus this was reassigned as “Aphanizomenonaceae Cluster 1” (Supplementary Figure S3). ASV1176 was assigned as “Microcystaceae” but phylogenetic inferences revealed this belonged to *Coelosphaerium* and was manually reassigned (Supplementary Figure S4). Three ASVs (ASV339, ASV751, ASV2762) were assigned as Microcystaceae and formed a well-supported clade away from known genera within the Microcystaceae, thus reassigned as “Microcystaceae Cluster 1” (Supplementary Figure S4). There were several sequences which were classified as Microcystaceae X, an undescribed genus within the Microcystaceae; this genus was within the top 15 most abundance genera (Figure 2). These ASVs formed a clade with no cultured strains (Supplementary Figure S4), only sequences which were collected in a culture-independent manner from other fresh waterbodies (e.g., Reelfoot Lake, Tennessee, United States). A phylogenetic tree of the picocyanobacteria, order Synechococcales, was constructed with 259 sequences, 176 of which were ASVs (Supplementary Figure S5).

Phylogenetic inferences of ASVs which could not be classified past the order level were also conducted. These were found in several clades across the tree (Supplementary Figure S6). Two ASVs (125 and 2,225) were only classified as Cyanophyceae class and manually reassigned as “Leptolyngbyaceae Cluster 1,” as these ASVs formed a clade within the Leptolyngbyaceae with sequences from other freshwater lakes. AVS’s 378 and 1838 were also only classified at the class level and fell within the Synechococcaceae and reassigned as “Synechococcaceae Cluster 1.” These ASV’s formed a well-supported clade with other uncultured sequences from freshwater bacterioplankton samples. ASV3311 was found to be *Pseudanabaena*, ASV5289 clustered with *Neocylindrospermum*, and several ASVs (ASV170, ASV437, ASV669, ASV793, ASV5103) clustered with *Nodosilinea*; these were all manually reassigned.

### 3.3. Cyanobacterial-bacterial correlations

**Figure 3:**
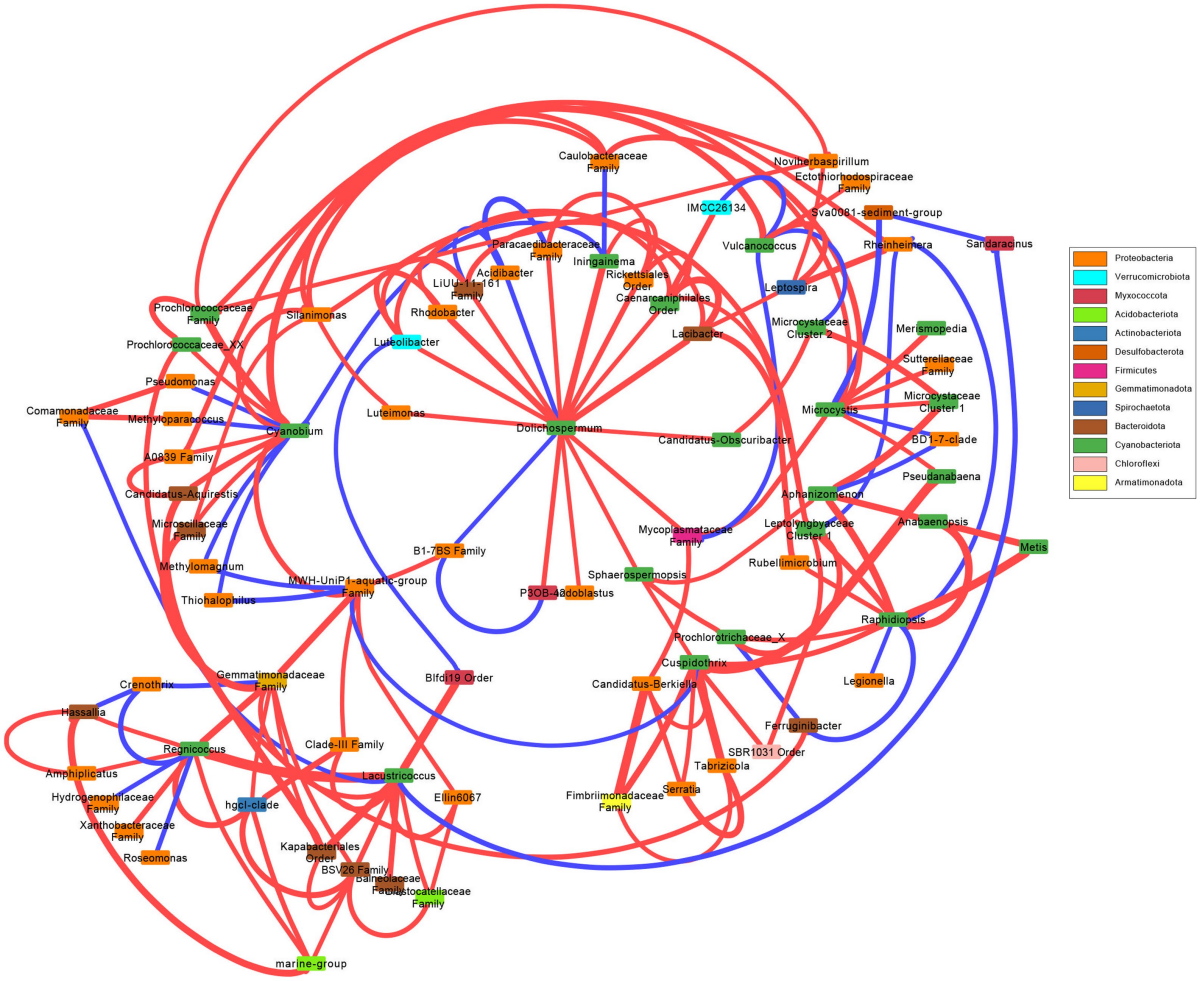
Network analysis of the most abundant cyanobacterial genera and the significant cyanobacterial-(cyano)bacterial relations. Edge thickness represent weight and color represent positive (red) or negative (blue) correlations. Box color represent phylum.

A network was created to observe the correlations between most abundant cyanobacterial genera (Figure 3). There were no correlated taxa, bacterial nor cyanobacterial, shared between *Dolichospermum*, *Microcystis*, and *Raphidiopsis*. *Dolichospermum* was positively correlated with several taxa, and negatively correlated with two bacteria including *Acidibacter*. *Microcystis* was correlated with less taxa than *Dolichospermum*, and negatively correlated with *Rheinheimera*. *Raphidiopsis* was also negatively correlated with *Rheinheimera*, in addition to *Legionella*. *Raphidiopsis* was correlated with several cyanobacterial taxa, in comparison to *Dolichospermum* and *Microcystis*. *Pseudanabaena* was correlated to both *Microcystis* and *Cuspidothrix*, however *Microcystis* and *Cuspidothrix* were not correlated with each other. The Prochlorococcecean taxa (i.e., *Cyanobium*, *Regnicoccus*, *Lacustricoccus*, *Vulcanococcus*) shared several correlated taxa, distinct from the crown cyanobacteria.

### 3.4. Community composition

**Figure 4:**
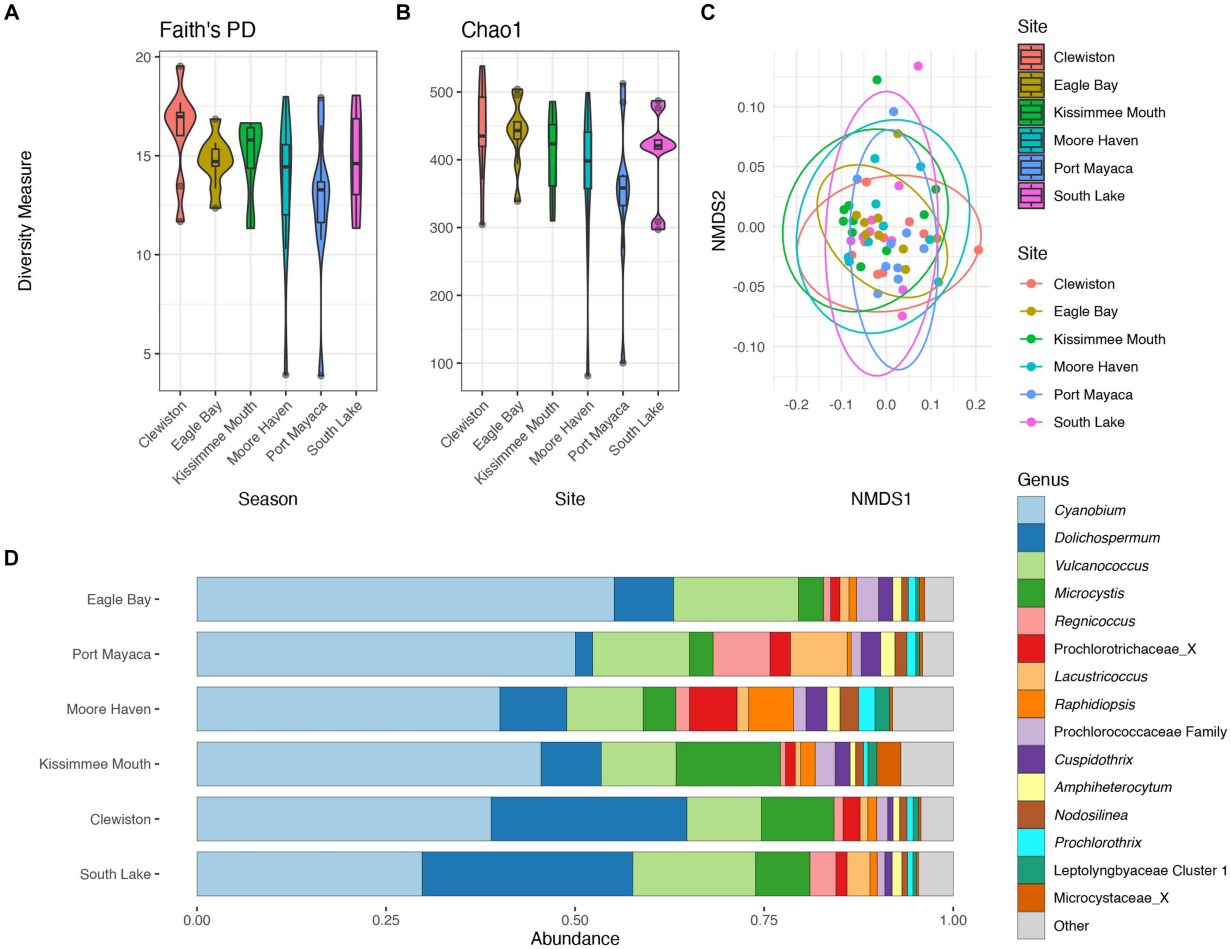
Diversity measures of the cyanobacterial communities at the different sampling locations. Faith phylogenetic diversity **(A)** and Chao1 index **(B)** between each sampling location. Non-metric Multidimensional Scaling (NMDS) ordination within two dimensions of the cyanobacterial communities based on generalized-Unifrac distances between sampling locations, colors represent **(C)**. Bar plot of the average relative abundance of the most abundant cyanobacterial genera at each location **(D)**.

There were no significant differences in taxonomic richness between cyanobacterial communities based on either metric at each sampling site (Figures 4A,B). The NMDS revealed overlap between these communities (Figure 4C), and results from the pairwise PERMANOVA showed that there were no significant differences in cyanobacterial communities between sites (*p* > 0.05). The southern region of the lake (i.e., Clewiston and South Lake) had higher relative abundances of *Dolichospermum*, while the northern region near the mouth of the Kissimmee River had a higher relative abundance of *Microcystis*; Moore Haven had the highest relative abundance of *Raphidiopsis* (Figure 4D).

Similar to the cyanobacterial communities, there were no significant differences in taxonomic richness between bacterial communities based on either metric at each sampling site (Supplementary Figures S7A,B). The NMDS revealed overlap between these communities (Supplementary Figure S7C), and results from the pairwise PERMANOVA showed that there were significant differences between bacterial communities between Clewiston and Kissimmee Mouth (R^2^ = 0.11, *p* = 0.03). There were no major differences between relative abundances of bacterial phyla between sites (Supplementary Figure S7D).

**Figure 5:**
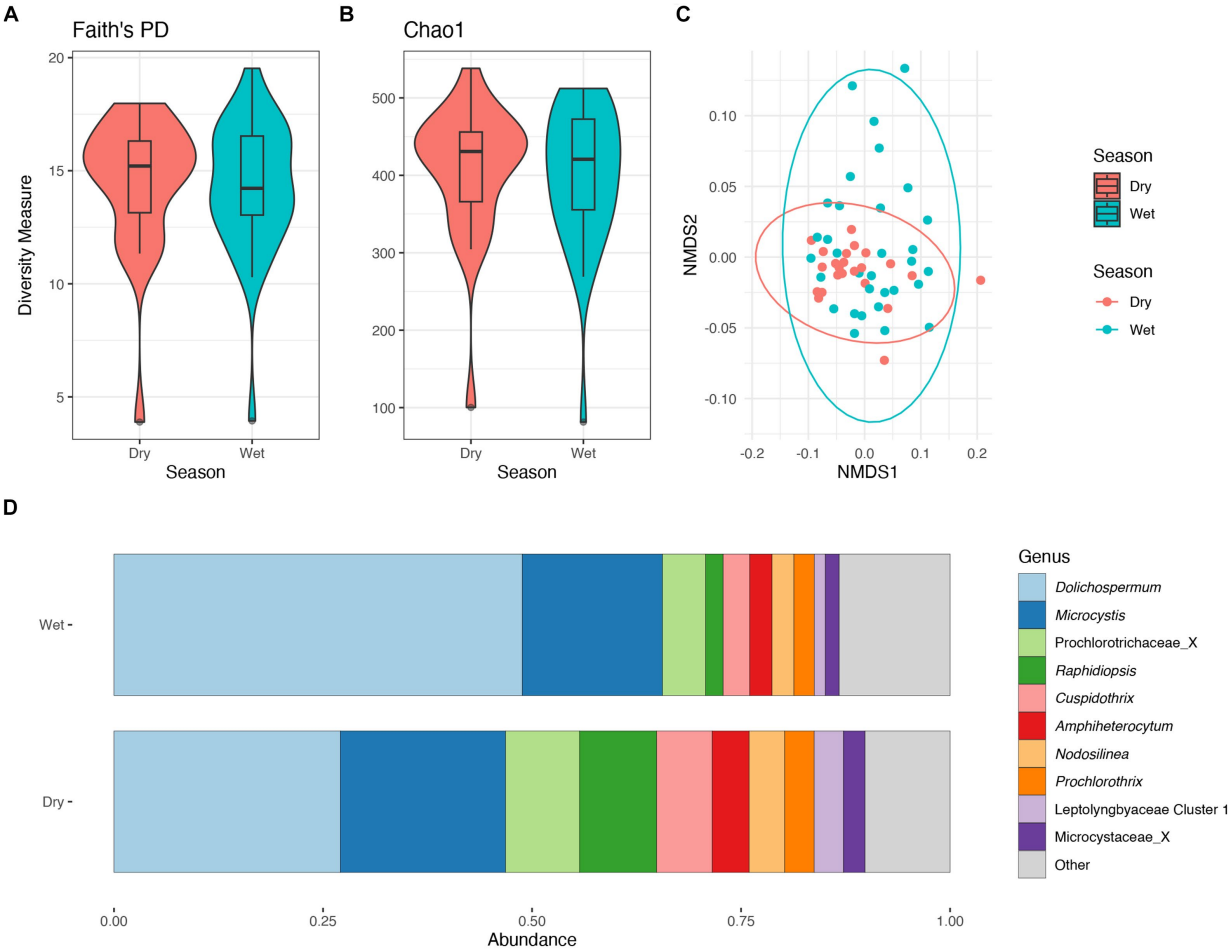
Diversity measures of the cyanobacterial communities between wet and dry season. Faith phylogenetic diversity **(A)** and Chao1 index **(B)** between each season. Non-metric Multidimensional Scaling (NMDS) ordination within two dimensions of the cyanobacterial communities based on generalized-Unifrac distances between sampling locations, colors represent season **(C)**. Bar plot of the average relative abundance of the most abundant, non-prochlorococcacean, cyanobacterial genera during each season **(D)**.

There were no significant differences in taxonomic richness in cyanobacterial communities between the wet and dry seasons (Figures 5A,B). The non-metric multidimensional scaling (NMDS) analysis showed an overlap between wet and dry seasons for the cyanobacterial communities (Figure 5C). Results from PERMANOVA showed significant differences between the cyanobacterial communities in the wet and dry seasons, although season accounted for a relatively small proportion of the variation data (R^2^ = 0.06, *p* < 0.01). The relative abundance of the Prochlorococcaceae was nearly equal between wet and dry seasons, accounting for ~60% of the cyanobacterial relative abundance (Supplementary Figure S8A). *Dolichospermum* relative abundance was nearly double in the wet season compared to the dry season, where it was also the dominant non-Prochlorococcaceae taxon. There also appeared to be an increased relative abundance in *Amphiheterocytum*, *Cuspidothrix*, and *Raphidiopsis* in the dry season; *Microcystis* relative abundance appeared even between the two seasons (Figure 5D). Indicator species, as genera, between seasonal communities were determined and listed in Supplementary Table S1.

There were no significant differences in taxonomic richness in bacterial communities between the wet and dry seasons (Supplementary Figures S9A,B). The non-metric multidimensional scaling (NMDS) analysis showed an overlap between wet and dry seasons for the cyanobacterial communities (Figure 5C). Results from PERMANOVA showed significant differences between the bacterial communities in the wet and dry seasons, although season accounted for a relatively small proportion of the variation data (R^2^ = 0.08, *p* < 0.001). There was an increased relative abundance of Proteobacteria (=Pseudomonadota) in the wet season, and an increased relative abundance of Actinobacteria (=Actinomycetota) in the dry season (Supplementary Figure S9D).

The relative abundance of the phylum Cyanobacteriota within the lake did not differ between seasons comprising ~30% of the relative abundance (Supplementary Figure S8C). Additionally, there were no significant differences in cyanobacterial communities between seasons based on alpha diversity metrics (Figures 5A,B). Conversely, the cyanobacterial communities differed between seasons based on results from the PERMANOVA.

**Figure 6:**
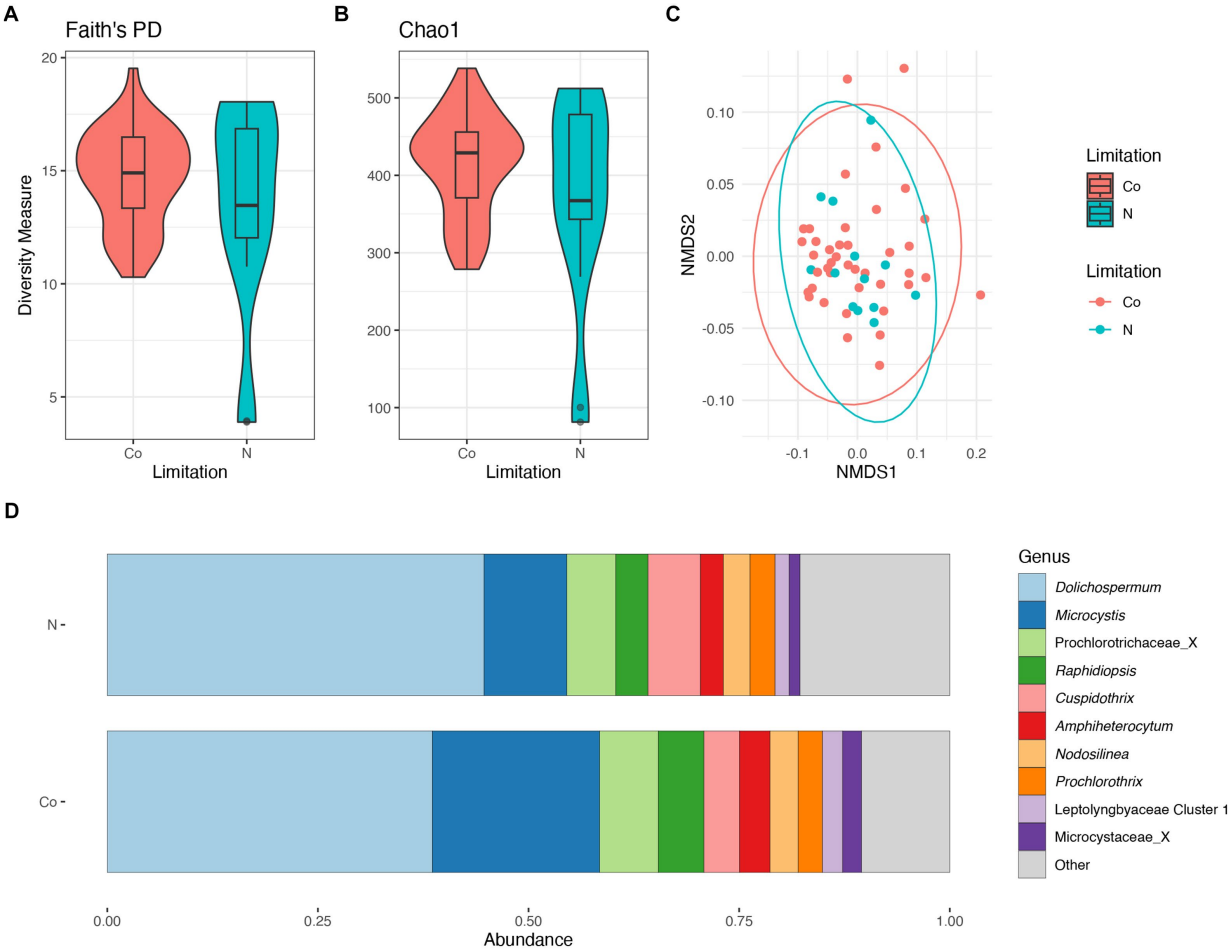
Diversity measures of the cyanobacterial communities between nitrogen and co-nutrient limitation. Faith phylogenetic diversity **(A)** and Chao1 index **(B)** between nutrient limitations. Non-metric Multidimensional Scaling (NMDS) ordination within two dimensions of the cyanobacterial communities based on generalized-Unifrac distances between nutrient limitations, colors represent limitation **(C)**. Bar plot of the average relative abundance of the most abundant, non-prochlorococcacean, cyanobacterial genera during nutrient limitations **(D)**.

There were no significant differences in taxonomic richness between cyanobacterial communities during N-limitation and co-limitation (Figures 6A,B). The NMDS analysis showed an overlap between the cyanobacterial communities during N-limitation and co-limitation (Figure 6C). When comparing the N-limited and co-limited cyanobacterial communities, there were no significant differences in community composition (PERMANOVA R^2^ = 0.02, *p* = 0.29). The relative abundance of the Prochlorococcaceae was nearly equal between N-limited and co-limited communities and accounted for ~55%–60% of the relative abundance, although their relative abundance was slightly higher in N-limited communities (Supplementary Figure S8B). When observing the non-Prochlorococcaceae taxa, there appears to be a non-significant increased relative abundance in *Dolichospermum* in the N-limited communities compared to the co-limited communities (PERMANOVA R^2^ = 0.02, *p* = 0.5), while *Microcystis* relative abundance showed the opposite trend (PERMANOVA R^2^ = 0.05, *p* = 0.2; Figure 6D). Indicator species, as genera, between N-limited and co-limited communities were determined; only *Planktothrix* and Synechococcaceae Cluster 1 were determined to be indicator species within N-limited communities. There was an increased relative abundance of cyanobacteria (=Cyanobacteriota) in the co-limited communities compared to the N-limited communities (Supplementary Figure S8D).

There were no significant differences in taxonomic richness between bacterial communities during N-limitation and co-limitation (Supplementary Figures S10A,B). The NMDS analysis showed an overlap between the bacterial communities in the wet and dry seasons (Supplementary Figure S10C). When comparing the N-limitation and co-limited cyanobacterial communities, there were no significant differences in community composition (PERMANOVA R^2^ = 0.01, *p* = 0.42). There were no major differences between relative abundances of any bacterial phyla between seasons (Supplementary Figure S10D). There was an increased relative abundance of Proteobacteria (=Pseudomonadota) and Actinobacteria (=Actinomycetota) in N-limited communities, and an increased relative abundance of Planctomycetota in the co-limited communities (Supplementary Figure S10D).

### 3.5. Influences of limnological parameters on common cyanobacteria

**Figure 7:**
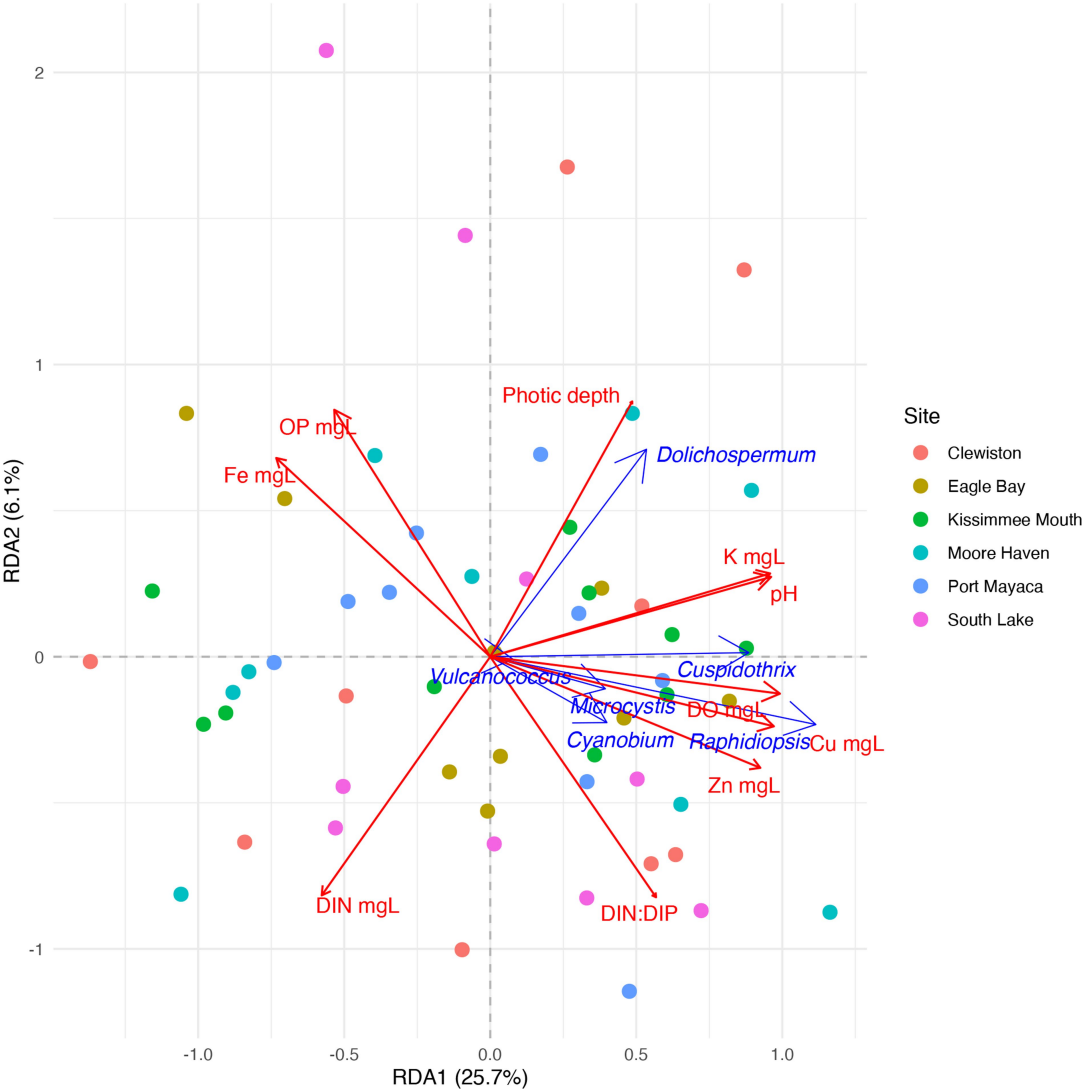
Partial redundancy analysis (pRDA) ordination within two dimensions of the cyanobacterial genera *Cuspidothrix*, *Cyanobium*, *Dolichospermum*, *Microcystis*, *Raphidiopsis*, and *Vulcanococcus*. Drivers of taxonomic variation are shown by blue arrows. Significant environmental drivers are shown by red arrows. Circles indicate samples, colors indicate sample site.

Due to the high relative abundance of *Cuspidothrix*, *Cyanobium*, *Dolichospermum*, *Microcystis*, *Raphidiopsis*, and *Vulcanococcus* (Figure 2) the environmental drivers of these taxa were subjected to further investigation via a partial redundancy analysis (pRDA). From the model, the conditioned variables (sample sites and outings) explained 11% of the variation, while water chemistry explained 34.4% of the variation, the remaining 54.6% of the variation was unexplained; the value of p for the model was 0.002. The triplot from the pRDA revealed that *Cuspidothrix, Cyanobium*, *Microcystis*, *Raphidiopsis*, and *Vulcanococcus* were associated with each other, but not *Dolichospermum* (Figure 7). *Cuspidothrix, Cyanobium*, *Microcystis*, and *Vulcanococcus* were positively associated with increased dissolved oxygen, copper, and zinc concentrations, and inversely associated with orthophosphate and iron concentration. *Dolichospermum* was positively associated with photic depth, inversely associated with DIN, and not associated with TRP.

**Figure 8:**
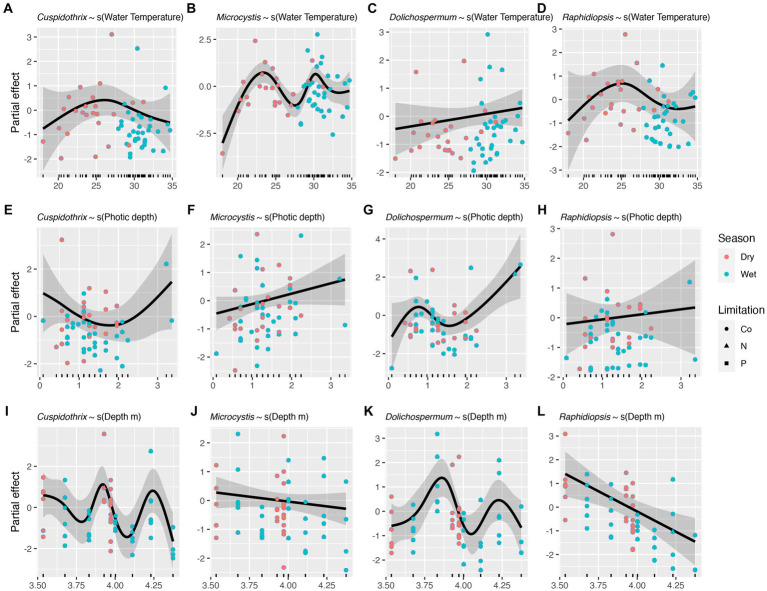
Effects of water temperature **(A–D)**, photic depth **(E–H)**, lake depth **(I–L)**, on bloom forming cyanobacteria as identified with generalized additive models (GAMs). Shaded areas indicate 95% confidence intervals, shapes indicate limitation, and colors indicate season.

**Figure 9:**
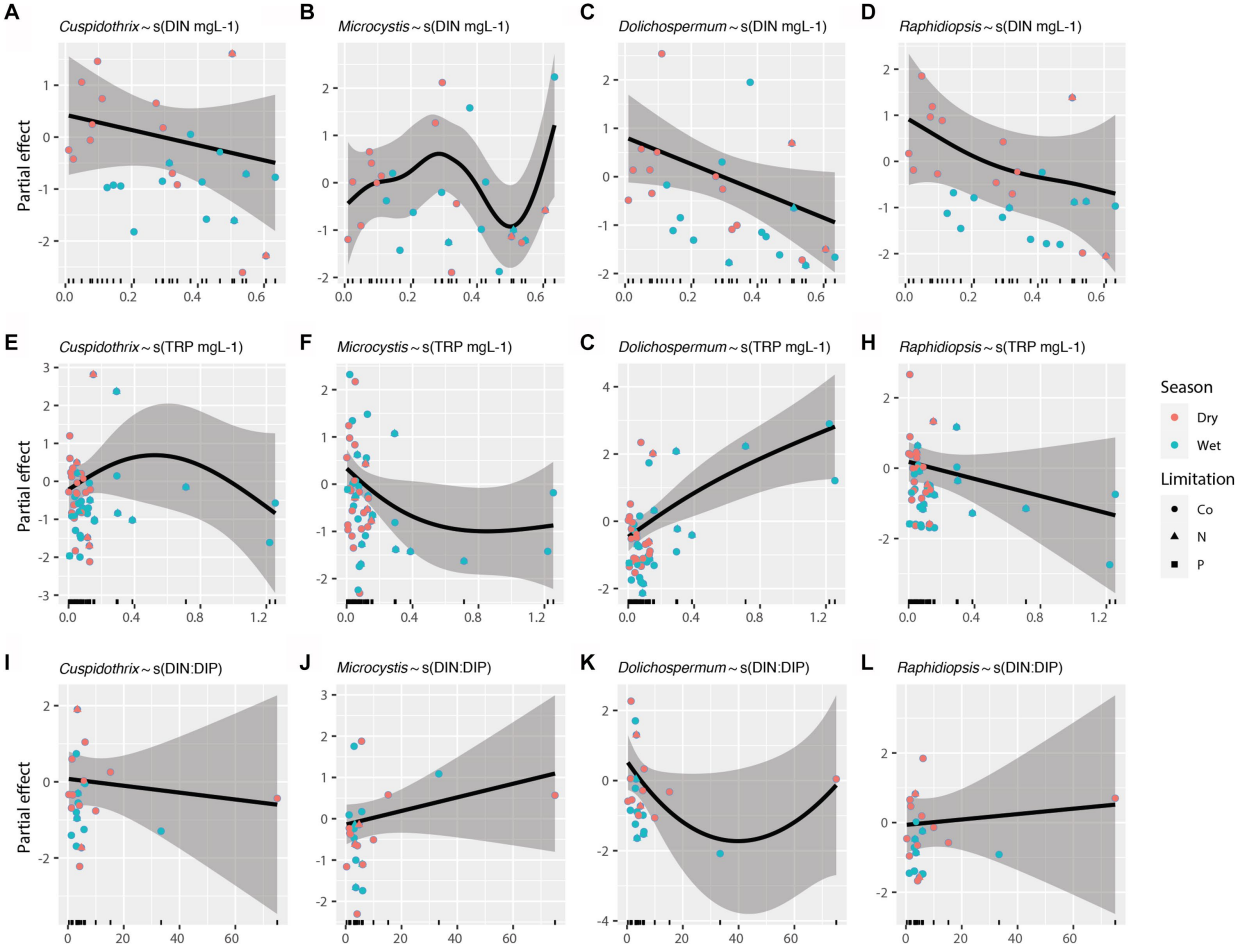
Effects of dissolved inorganic nitrogen **(A–D)**, total reactive phosphorus **(E–H)**, and DIN:DIP **(I–L)** on bloom forming cyanobacteria as identified with generalized additive models (GAMs). Shaded areas indicate 95% confidence intervals, shapes indicate limitation, and colors indicate season.

Effects of individual limnological parameters (i.e., water temperature, photic depth, lake depth, DIN, TRP, and DIN:DIP) on *Cuspidothrix*, *Cyanobium*, *Dolichospermum*, *Microcystis*, *Raphidiopsis*, and *Vulcanococcus* relative abundances were investigated using GAMs. In this study, GAMs used the negative binomial distribution assumption; sample outing and site were regarded as random effects. Results are listed in Table 1. The effects of water temperature, photic depth, lake depth, and conductivity on bloom-forming genera (i.e., *Cuspidothrix*, *Dolichospermum*, *Microcystis*, and *Raphidiopsis*) are visualized in Figure 8 and the relationship between nutrients and bloom-forming cyanobacteria are visualized in Figure 9. The diazotrophic bloom-forming genera (i.e., *Cuspidothrix*, *Dolichospermum* and *Raphidiopsis*) were inversely correlated with increases in DIN (Figures 9A,C,D; Table 1), whereas *Microcystis* was positively correlated with DIN (Figure 9B; Table 1). *Dolichospermum* was positively, and significantly, correlated with increased TRP concentrations whereas *Raphidiopsis* relative abundance decreased with increasing TRP concentrations, although this trend was not significant (Figures 9F,H; Table 1). *Cuspidothrix* relative abundance was positively correlated with TRP concentrations up until ~0.5 mgL^−1^ after which it began to decrease (Figure 9E; Table 1). There was no noticeable relationship between *Microcystis* relative abundance and TRP concentrations (Figure 9F; Table 1), Both *Cuspidothrix* and *Dolichospermum* relative abundances were negatively correlated with DIN:DIP, whereas *Microcystis* and *Raphidiopsis* relative abundances were positively, and linearly, correlated with DIN:DIP (Figures 9I–L; Table 1). Water temperature had a varied response on the bloom-forming genera, with *Cuspidothrix* and *Raphidiopsis* having the highest relative abundances in the cooler waters (~25°C) of the dry season, whereas *Dolichospermum* and *Microcystis* relative abundances increased with increasing water temperatures (Figures 8A–D). Both *Dolichospermum* and *Microcystis* relative abundances peaked around 30°C, in the warmer wet season. *Dolichospermum* and *Microcystis* relative abundances were positively correlated with photic depth (Figures 8E–H; Table 1), whereas there were no trends between *Cuspidothrix* and *Raphidiopsis* relative abundances and photic depth. Furthermore, lake depth had a significant negative linear relationship with *Raphidiopsis* relative abundance (Figure 8L; Table 1).

**Table 1:** Results of the generalized linear models (GAMs).

Genus	Explanatory variable	edf	Deviance %	R^2^	AIC
*Cuspidothrix*	s(Water Temperature)	2.1	27.4	0.049	508
	s(Photic Depth m)	2.4	30.6	0.032	505
	s(Lake Depth m)^**^	6.1	45.5	0.043	497
	s(DIN mg L^−1^)^*^	1	43.5	0.04	231
	s(TRP mg L^−1^)	4.2	44.6	0.03	436
	s(DIN:DIP)	1	49.5	−0.314	208
*Cyanobium*	s(Water Temperature)	1	1.42	0.006	831
	s(Photic Depth m)^*^	2.6	12.2	0.065	828
	s(Lake Depth m)	1	5.97	0.056	829
	s(DIN mg L^−1^)	1	5.04	0.07	389
	s(TRP mg L^−1^)^**^	1	9.31	0.101	725
	s(DIN:DIP)	1	0.038	−0.044	350
*Dolichospermum*	s(Water Temperature)	1	21	0.047	685
	s(Photic Depth m)^**^	3.4	45.1	0.577	667
	s(Lake Depth m)^**^	4.9	42.4	0.191	671
	s(DIN mg L^−1^)^*^	1	9.45	−0.011	313
	s(TRP mg L^−1^)^***^	1.2	41.5	0.479	582
	s(DIN:DIP)	1.7	18.5	0.012	278
*Microcystis*	s(Water Temperature)	4.6	36.3	0.123	616
	s(Photic Depth m)^	1	14	0.452	627
	s(Lake Depth m)	1	13	0.036	628
	s(DIN mg L^−1^)	4.2	40.8	0.255	304
	s(TRP mg L^−1^)^	1.7	20.4	0.066	552
	s(DIN:DIP)	1	37.9	0.378	269
*Raphidiopsis*	s(Water Temperature)	2.6	12.8	0.033	439
	s(Photic Depth m)	1	3.21	−0.008	441
	s(Lake Depth m)^**^	1	14.2	0.072	425
	s(DIN mg L^−1^)	1.4	11.1	−0.08	184
	s(TRP mg L^−1^)	1	5.45	−0.001	394
	s(DIN:DIP)	1	13	−1.5	168
*Vulcanococcus*	s(Water Temperature)	1	19.9	0.15	689
	s(Photic Depth m)	1	13	0.09	694
	s(Lake Depth m)	1	15.6	0.116	691
	s(DIN mg L^−1^)	1	0.166	−0.038	324
	s(TRP mg L^−1^)	1.6	16.9	0.118	611
	s(DIN:DIP)	1	8.96	0.046	286

Significant explanatory variables are designated by *p* < 0.1 (^), *p* < 0.05 (*), *p* < 0.01 (**), *p* < 0.001 (***).

The effects of water temperature, photic depth, lake depth, and conductivity on *Cyanobium* and *Vulcanococcus* are visualized in Supplementary Figure S11 and the effects of nutrients in Supplementary Figure S12. Water temperature had a positive linear relationship on both *Cyanobium* and *Vulcanococcus* relative abundance, although this relationship was greater on *Vulcanococcus* (Supplementary Figures S11A,D; Table 1). Both *Cyanobium* and *Vulcanococcus* relative abundance had a negative relationship with both photic and lake depth (Supplementary Figures S11B,C,E,F; Table 1). DIN had a negative, although weak, correlation with *Cyanobium* and *Vulcanococcus* relative abundance (Supplementary Figures S12A,D; Table 1). TRP had a strong negative correlation with *Cyanobium* relative abundance (Supplementary Figure S12B; Table 1). DIN:DIP had no relationship with *Cyanobium* relative abundance, but had a negative, and linear, relationship with *Vulcanococcus* relative abundance (Supplementary Figures S12C,F; Table 1).

### 3.6. Cyanotoxins

Cyanotoxins were detected on 16 occasions over the annual cycle at the six sites sampled. Microcystins, nodularins, and anatoxin-a were detected throughout the lake and through time (Supplementary Table S2). Both microcystin-LR (MC-LR) and microcystin-RR (MC-RR) were detected with MC-LR being the most commonly occurring congener; MC-LR was detected 11 times and MC-RR once. Nodularins were detected seven times, and anatoxin-a was detected once. Microcystin-LR co-occurred with nodularins three times. Cyanotoxin concentrations were low, and ranged between 0.04 and 1.4 μg L^−1^, with nodularin being the toxin with the highest concentration (1.12 μg L^−1^), while MC-LR reached concentrations of 0.6 μg L^−1^. Cyanotoxins were detected nine times during the wet season in four out of six sampling events and seven times in the dry season in three out of four sampling events. Anatoxin-a was only detected once, in the wet season. Nodularins occurred more frequently in the dry season than the wet season (five vs. two occurrences) while microcystins were observed six times in the wet season and five times in the dry season. Due to the infrequent occurrence of these toxins, statistical analyses to elucidate drivers of their occurrence proved unsuccessful (data not shown).

Several known toxin producing genera occurred (e.g., *Aphanizomenon*, *Dolichospermum*, *Microcystis*, *Raphidiopsis*). A correlation analyses was applied to identify which genera were correlated with these cyanotoxins (Supplementary Figure S13). There were several genera correlated with MC-LR, with *Microcystis* being the only confirmed microcystin producer in Lake Okeechobee. Several genera were correlated to MC-RR, with *Chrysosporum*, *Microcystis*, and *Planktothricoides* being the known toxin producing genera. *Aphanizomenon*, *Lagosinema*, Microcystaceae Cluster 1, *Parasynechococcus*, *Planktothricoides*, Prochlorococcaceae_XX, and *Pseudanabaena* were the genera most correlated with nodularins (Supplementary Figure S13).

## 4. Discussion

This study provides a detailed analysis of Lake Okeechobee’s spatiotemporal cyanobacterial and bacterial community structure. Lake Okeechobee has gained notoriety for its *Microcystis*-dominated cyanoHABs in the past decade. Until now, characterizations of the cyanobacterial community structure in Lake Okeechobee have been carried out via microscopy (e.g., Havens et al., 1994; Cichra et al., 1995; Beaver et al., 2013), apart from Kramer et al. (2018) which focused on full metagenomic sequencing of the cyanoHAB that occurred in 2016. Thus, few data exist on molecular characterizations of the bacterial/cyanobacterial community structure within Lake Okeechobee and this study is the first of its kind.

### 4.1. Cyanobacterial diversity and cyanotoxins

Many of the cyanobacterial taxa that are well documented in the literature via microscopy (e.g., *Aphanizomenon*, *Dolichospermum* [=*Anabaena*], *Microcystis,* and *Raphidiopsis* [=*Cylindrospermopsis*]) were identified from the molecular methods employed in this study. Surprisingly, the high abundance of Prochlorococcacean cyanobacteria was not expected, as these taxa are not well recorded in Lake Okeechobee, likely due to their small size (< 2 μm). Additionally, the cyanobacterial genus *Planktolyngbya*, whose presence in Lake Okeechobee is well documented (e.g., Beaver et al., 2013), was not observed in the molecular data. However, *Limnolyngbya* was observed which was separated from *Planktolyngbya* (Li and Li, 2016), and may be the correct taxon. Within the Aphanizomenonaceae a single ASV (176) clustered with sequences classified as *Anabaena* and *Dolichospermum* but away from these genera (Supplementary Figure S3). Within the Microcystaceae, the ASVs labeled as Microcystaceae Cluster 1, clustered with sequences from the freshwater lakes, Las Cumbres Lake (Panama) and Reelfoot Lake (Tennessee, United States), and may represent a widespread cyanobacterium (Supplementary Figure S4). Phylogenetic inferences of the ASVs that could not be classified past the class level revealed potentially novel cyanobacterial diversity within the lake. There were two clades of ASV’s which clustered within the Leptolyngbyaceae and Synechococcaceae, respectively, which may represent novel diversity (Supplementary Figure S6).

Picocyanobacteria (<2 μm) belonging to the family Prochlorococcaceae dominated the cyanobacterial community throughout the lake, with *Cyanobium* demonstrating the highest relative abundance followed by *Vulcanococcus*. The genera *Dolichospermum*, *Microcystis*, and *Raphidiopsis* are known to cause cyanoHABs within Lake Okeechobee (e.g., Jones, 1987; James et al., 2008; Kramer et al., 2018) and were highly abundant throughout the lake during this study (Figure 2). Other bloom-forming, diazotrophic *Aphanizomenon*-like and *Dolichospermum*-like genera, such as *Cuspidothrix* and *Sphaerospermopsis*, were also observed in the molecular data, although their presence in Lake Okeechobee have not been recorded, likely due to their cryptic morphology (Rajaniemi et al., 2005; Werner et al., 2012).

Notably, *Dolichospermum* was not correlated to microcystins nor to nodularins (Supplementary Figure S13). *Dolichospermum* is known to produce several cyanotoxins (Otten and Paerl, 2015), however toxin production by *Dolichospermum* within Lake Okeechobee remains unknown, although metagenomic analyses suggest it may produce saxitoxin (Kramer et al., 2018). From the correlation analysis, the potential producer of nodularins remains obscure as none of the positively correlated genera are known producers of nodularins (Supplementary Figure S13). Conversely, *Iningainema* is an established nodularin producer (McGregor and Sendall, 2017; Berthold et al., 2021) which is known to occur in the lake (Laughinghouse lab, unpublished data), however its abundance was negatively correlated to nodularin concentrations (Supplementary Figure S13). *Microcystis* is a known microcystins producer within Lake Okeechobee (Lefler et al., 2020; Kinley-Baird et al., 2021) and is the likely toxin producer, although other taxa may also be producing these toxins. The correlations between the picocyanobacteria genera and toxins (i.e., *Parasynechococcus* with MC-LR, and Prochlorococcaceae_XX with nodularin) were unexpectedly high considering the diversity of the known toxigenic taxa (Supplementary Figure S13). While this group are not traditionally considered toxigenic, it has been recently found that picocyanobacteria in tropical freshwaters are capable of cylindrospermopsin production (Gin et al., 2021; Sim et al., 2023). Thus, it imperative to further assess the toxigenic potential of these abundant cyanobacteria. The lack of definitive correlations between a genus (or genera) and toxins highlights the unknown toxigenic potential within the lake.

The community structure was more variable during the wet season, while communities from the dry season were more similar (Figure 5C). Communities within both seasons were dominated by picocyanobacteria (e.g., *Cyanobium*) with an non-significant increase in *Dolichospermum* in the wet season (PERMANOVA R^2^ = 0.02, *p* = 0.2), and significant increase in *Raphidiopsis* in the dry season (PERMANOVA R^2^ = 0.08, *p* = 0.02; Figure 5D). *Raphidiopsis* relative abundance was higher in the dry season (Figure 5D) and determined to be an indicator species for dry season communities (Supplementary Table S1). Furthermore, *Raphidiopsis* relative abundance was higher at Moore Haven, the headwaters of the Caloosahatchee River, a shallow area of the lake within the rim canal. While *Raphidiopsis* blooms are uncommon in Lake Okeechobee, *Raphidiopsis*-dominated blooms have recently been observed in the shallow areas of the lake (i.e., transition zone) during dry periods (Laughinghouse and Lefler, pers. observ.). The communities at the mouth of the Kissimmee River had a higher relative abundance of *Microcystis* in comparison to other locations (Figure 4D). This region of Lake Okeechobee is known to have increased frequency of *Microcystis*-dominated cyanoHABs, likely due to external nutrient loading from the Kissimmee drainage basin (Havens et al., 1994). Conversely, the southern region of the lake is distant from major inflows and thus external nutrient loadings and was found to possess higher relative abundance of *Dolichospermum* (Figures 2, 4D).

### 4.2. Drivers of abundant cyanobacterial genera

Increases in both N and P are known to drive the growth of bloom-forming genera, although their concentrations and ratios have disparate effects on these genera (Paerl et al., 2016). Our results highlight the disparate responses in abundances of these bloom-forming genera to N, P, and DIN:DIP. While increasing DIN concentrations had a negative relationship on relative abundance of the bloom-forming diazotrophic genera, as expected, only *Dolichospermum* relative abundance had a positive, and linear, response to increasing TRP concentrations (Figures 9E,G,H). *Cuspidothrix* relative abundance was highest with TRP concentrations around ~0.5 mg L^−1^ (Figure 9E), potentially indicating this genus has lower P requirements, but higher than that of *Raphidiopsis*. These data suggest that P concentrations do not affect all bloom-forming diazotrophic genera similarly. Furthermore, *Raphidiopsis* relative abundance had an increased, although weak, response to DIN:DIP, whereas *Cuspidothrix* and *Dolichospermum* relative abundances were negatively correlated with increasing DIN:DIP (Figures 9I,K,L), suggesting that DIN:DIP, and potentially TN:TP, does not affect all diazotrophs equally. *Dolichospermum* and *Microcystis* relative abundances were positively affected by photic depth (Figures 8F,G; Table 1), supporting previous research on their drivers within Lake Okeechobee (Havens et al., 1998, 2003). *Cuspidothrix* and *Raphidiopsis* relative abundances were both generally unaffected by photic depth, indicating these genera are more adapted to low light conditions, an observation supported by previous research on *Raphidiopsis* within the system (Havens et al., 2003).

Altogether, these genera have overlapping, but distinct niches within Lake Okeechobee. *Dolichospermum* and *Microcystis* favor the warmer wet season, in clear waters with increased photic depth, which align with previous research on the lake (Havens et al., 1998). They differ in their nutrient requirements, with *Dolichospermum* benefitting from waters with low DIN and high TRP concentrations whereas *Microcystis* is ambivalent to TRP concentrations and prefers waters with a high DIN:DIP (Paerl et al., 2016; Chia et al., 2018). *Cuspidothrix* and *Raphidiopsis* were more prevalent during the cooler waters of the dry season with low DIN, where *Cuspidothrix* relative abundance is correlated with higher photic depths and *Raphidiopsis* is correlated with shallow waters.

Spatially, *Dolichospermum* relative abundance was highest in the southern region of the lake (i.e., South Lake and Clewiston), away from sites of significant hydrological, and thus external nutrient, inputs. This genus prefers increased SRP concentrations and likely benefits from the internal legacy P that is continuously resuspended and released from the sediments. Furthermore, the lack of an external N load likely behooves *Dolichospermum* in so far as reducing competition from non-diazotrophic bloom forming genera, specifically *Microcystis*. *Microcystis* relative abundances was highest in the northern part of the lake, at the mouth of the Kissimmee River, which is known to have an increased abundance of *Microcystis* (Havens et al., 1994). Kramer et al. (2018) indicated that increases in N concentrations in Lake Okeechobee promotes non-diazotrophic cyanobacterial abundance in the lake and data from Zhang et al. (2011) show a significant increase in TN concentrations from several point sources in this region of the watershed. The increase in *Microcystis* abundance in the northern region is likely due to the N rich inputs from the Kissimmee drainage basin via the Kissimmee River, which accounts for ~70% of the inputs into Lake Okeechobee (Zhang J. et al., 2020), as well as the surrounding agricultural and urban inputs. This area has a large drainage basin that is affected by both agricultural and urban runoff, contributing to nutrient inputs (e.g., N & P). Modern fertilizers are comprised of ammonium and urea as their source of nitrogen and their increased use is hypothesized to drive harmful algal blooms, known as the HAB-HB (Harmful Algal Bloom-Haber Bosch) connection (Glibert et al., 2014); additional sources of urea include sewage/septic and livestock runoff. Urea can represent >50% of the dissolved organic nitrogen pool and can be high in agriculturally impacted lakes (Bogard et al., 2012). An increase in urea may give *Microcystis* a competitive advantage as it is capable of assimilating urea as a source of carbon and nitrogen (Krausfeldt et al., 2019). However, neither total nitrogen nor organic nitrogen were quantified during this study and, to the authors best knowledge, urea concentrations in Lake Okeechobee are unknown. The effects of urea, and other forms of organic nitrogen, on *Microcystis* abundance in Lake Okeechobee remain unknown and warrants further investigation to better understand bloom drivers in this system.

Considering the lake is an N, or co-nutrient, limited system, nitrogen inputs also likely lead to an increase in N:P. During this study, the northern region was frequently co-nutrient limited, experiencing N-limitation only briefly (Supplementary Figure S1). Together these results, along with results from Kramer et al. (2018), suggest N inputs, both organic and inorganic, from the Kissimmee River can promote increased *Microcystis* abundance in the northern region of the lake, potentially effectuating *Microcystis* blooms throughout Lake Okeechobee. Furthermore, there is a need to understand the role of organic N (e.g., urea) within this system to determine how much is coming into the lake and its effect on *Microcystis* and other bloom forming taxa.

In comparison to the bloom-forming genera, the drivers of the picocyanobacteria are more elusive. Notably, *Vulcanococcus* relative abundance was positively correlated with water temperatures, and decreased with increased DIN:DIP (Supplementary Figures S11A, S12C), whereas the relative abundance of *Cyanobium* decreased with increasing TRP (Supplementary Figure S12B). The picocyanobacteria likely dominate the system due to their large surface-to-volume ratio that facilitates nutrient uptake when nutrients are scarce and reduces their light requirements (Havens et al., 1998).

Since the taxonomic resolution of metabarcoding is limited, the potential drivers are for the genera, and are not species specific. A total of six ASVs corresponded to *Microcystis* (Supplementary Figure S4), which may be several different species of *Microcystis*. *Microcystis* species are known to form microcystin-producing blooms within Lake Okeechobee (Kinley-Baird et al., 2021; Pokrzywinski et al., 2022), however both microcystin and non-microcystin producing *Microcystis* species are known to occur and bloom in Florida (Lefler et al., 2020, 2022, 2023). Furthermore, recent phylogenomic analyses supported several of the morphologically different species of *Microcystis*, each with various toxigenic potential (Cai et al., 2023). Similarly, several species of *Dolichospermum* and *Raphidiopsis* are known to occur in Lake Okeechobee (Cichra et al., 1995; Havens et al., 2003). Due to the diversity of bloom-forming genera within Lake Okeechobee, it is imperative to characterize these taxa and experimentally test how limnological parameters (e.g., N, P, water temperature, etc.) affect their potential to bloom and synthesize various toxins using *in-situ*, *ex-situ*, and strain level approaches (e.g., Kramer et al., 2018; Wagner et al., 2021).

### 4.3. Cyanobacterial-bacterial relationships

Cyanobacterial-bacterial relationships have garnered large interest in the past years (Morris et al., 2011; Cook et al., 2020; Smith et al., 2021), highlighting the importance of these enigmatic relationships. These relationships can be mutualistic (Woodhouse et al., 2016), cyanoHABs can alter the bacterioplankton communities (Berry et al., 2017), and some cyanobacterial genera (e.g., *Microcystis*) can be dependent on bacteria, and cyanobacteria, within their mucilage (Cook et al., 2020). Relationships between *Microcystis* and bacteria have been the focus of much research (e.g., Cook et al., 2020; Smith et al., 2022). However, these studies have concentrated on the relationships between *Microcystis* and the epibiont bacterial communities (e.g., Cook et al., 2020; Smith et al., 2021) or understanding the distinctions between epibiont and pelagic bacterial communities during bloom conditions (e.g., Parveen et al., 2013; Louati et al., 2023). However, temporal associations between cyanobacterial and co-occurring bacterioplankton during non-bloom conditions are rarely investigated. Previous research on Lake Taihu (China), another shallow subtropical lake, indicated that the bacterial community structure changes with the phytoplankton community (Niu et al., 2011).

Overall, the bacterial and cyanobacterial communities changed concurrently, with significant differences between communities in the wet and dry seasons, with neither cyanobacterial nor bacterial communities differing between N or co-limitation. The majority of the associated bacterial taxa belonged to the Proteobacteria (=Pseudomonadota; Figure 3), which was the dominate bacterial phylum, excluding Cyanobacteriota (Supplementary Figures S8C,D). Of the bloom forming genera *Cuspidothrix*, *Dolichospermum*, *Microcystis*, and *Raphidiopsis*, only *Cuspidothrix* and *Raphidiopsis* were correlated. Furthermore, only a single bacterial taxon, an unknown member of the Firmicutes (=Bacillota) was shared between *Dolichospermum* and *Microcystis*, this lack of correlated bacteria is supported by results from Louati et al. (2015) (Figure 3). *Cuspidothrix* and *Raphidiopsis* both occupied a similar niche within Lake Okeechobee and were correlated with each other but shared no correlated taxa. However, *Cuspidothrix* and *Microcystis* occupied distinct niches within Lake Okeechobee, and were not correlated with each other, but were both positively correlated with the cyanobacterial genus *Pseudanabaena* (Figure 3). This genus is known to occur within the mucilage of *Microcystis* colonies and may occupy the sheath of *Cuspidothrix*. Despite the dominance of *Cyanobium*, this genus was not correlated to the common bloom-forming genera, *Cuspidothrix*, *Dolichospermum*, and *Raphidiopsis*, but was correlated with *Microcystis* (Figure 3).

*Dolichospermum* shared correlations with several bacteria (*n* = 11) across five phyla, compared to *Microcystis*’ two (Figure 3). *Cuspidothrix* was also correlated to several bacteria (*n* = 4) across three phyla. This may indicate that *Cuspidothrix* and *Dolichospermum* have coevolved with these bacteria and/or have a symbiotic relationship. Conversely, *Raphidiopsis* was only positively correlated to a single bacteria genus, *Rubellimicrobium*, a member of the Pseudomonadota, and may not be as reliant on bacterial interactions as *Dolichospermum*. However, more in-depth analyses (e.g., *in situ* metagenomic and metatranscriptomic studies, laboratory experiments) are needed to better understand these relationships (e.g., Vico et al., 2021; Zuo et al., 2022).

In addition to other cyanobacteria, *Microcystis* was only positively correlated to Pseudomonadota, with its sole negative correlation with an unknown member of the Desulfobacterota (Figure 3). Proteobacteria are known to co-occur with *Microcystis* blooms (Parveen et al., 2013; Louati et al., 2023) and are copiotrophic (Simonato et al., 2010). One of the known correlated genera within the Pseudomonadota was *Silanimonas*. *Silanimonas* is known to co-exist with *Microcystis*, and the species *S. algicola* was isolated from a *Microcystis* colony (Chun et al., 2017). This species is known to perform nitrate reduction, part of the denitrification process in aerobic systems. However, it remains to be seen if denitrification was occurring, although denitrification is known to occur during cyanoHABs (Chen et al., 2011; Zhang et al., 2017).

In contrast to the bloom-forming genera, the picocyanobacteria (*Cyanobium*, *Lacustricoccus*, and *Regnicoccus*) were correlated with several bacteria and cyanobacteria (Figure 3). Whereas *Vulcanococcus* possessed few correlated taxa, many of which were negative. Similarly, to the closely related *Prochlorococcus*, these freshwater picocyanobacteria are likely reliant on co-occurring bacterial taxa (Morris et al., 2012).

Understanding the complex relationships between bacteria and cyanobacteria has the potential to provide valuable insights into functional bacterial traits that may assist cyanobacterial bloom proliferations (Louati et al., 2023). Due to the limitations of these data (i.e., 16S rRNA), those potential functional roles of the co-occurring pelagic bacterial taxa cannot be assessed; however, future efforts should be mindful of these relationships. Additionally, due to the shallow depth and polymictic nature of Lake Okeechobee, we cannot rule out if some of these bacterial taxa are particle-associated, and resuspended sediment particles. Furthermore, this study was limited to the bacterial community, and relationships with protists and other microalgae were not studied and may help further elucidate these dynamics. These data do, however, highlight an important observation that within the same water body, disparate bloom-forming cyanobacteria co-occur with dissimilar associated bacterial taxa.

## 5. Conclusion

These data highlight the cyanobacterial diversity within Lake Okeechobee, confirming the presence of many genera found from previous morphological assessments of the cyanobacteria, as well as highlighting potentially novel cyanobacteria. *Cyanobium* dominates the cyanobacterial communities, with *Cuspidothrix*, *Dolichospermum*, *Microcystis*, *Raphidiopsis*, and *Vulcanococcus* as the most abundant described genera. There were no differences between cyanobacterial nor bacterial communities during N or co-nutrient limitation. The cyanobacterial and bacterial communities significantly differ between wet and dry season, with a significant increase in *Raphidiopsis* in the dry season, although both seasons showed dominance of the picocyanobacteria. *Cuspidothrix*, *Dolichospermum*, *Microcystis*, and *Raphidiopsis* were the most commonly occurring bloom-forming taxa and possessed contrasting environmental drivers and microbial communities. Overall, these three bloom-forming genera have distinct abiotic drivers within Lake Okeechobee. Both *Dolichospermum* and *Microcystis* prefer the warmer wet season, in waters with increased photic depth. Our results, along with others (i.e., Havens et al., 1994; Kramer et al., 2018), suggest N inputs, both organic and inorganic, from the Kissimmee River can promote *Microcystis* abundance in the northern region of the lake, potentially effectuating *Microcystis* blooms in Lake Okeechobee. Furthermore, there is a need to understand the effects of organic forms of N (e.g., urea) on *Microcystis* in this system to elucidate whether it’s a species of nitrogen (e.g., NO_3_, urea, etc.) or solely the N:P ratio which may drive these proliferations. *Dolichospermum* increased abundance in the southern region of the lake, away from external nutrient inputs, may indicate that it benefits from resuspension of legacy P from sediments from the increased weather events associated with the wet season. In addition to their disparate abiotic drivers, these two genera were correlated with distinct bacteria, which may facilitate their ability to form blooms and out-compete one another, or other, cyanobacteria when conditions are right. *Cuspidothrix* and *Raphidiopsis* relative abundance increases in the cooler waters of the dry season, with low DIN and TRP concentrations. While these genera are correlated (Figure 3), and have similar nutrient requirements (Figure 9), they differ by lake depth, with *Raphidiopsis* relative abundance higher in shallow waters. Additionally, *Cuspidothrix* and *Raphidiopsis* have distinct correlated bacteria, which may promote dominance of one over the other when abiotic conditions are ideal for both.

These data highlight the variable nutrient requirement and niches these bloom-forming genera occupy within Lake Okeechobee, increasing our understanding of who is blooming when and why. They also highlight the need for dual nutrient control, as increases in both N and P can drive different bloom-forming genera.

## Data availability statement

Sequences were deposited at the Sequence Read Archive of the National Center for Biotechnology Information (NCBI) and made publicly available under accession number PRJNA967631. R code used for data analysis, including a full list of R packages, is on GitHub (github.com/flefler/LakeOkeechobee_16SrRNA).

## Author contributions

FL, DB, and HL contributed to the conception and design of the study and finalized the manuscript. FL, MB, DB, and HL collected data. FL, MB, DB, and PZ were responsible for the laboratory data analyses. FL analyzed sequencing data, performed statistical analyses, and wrote the first draft of the manuscript. HL supervised the project and secured funding. FL, MB, DB, PZ, AS, and HL critically reviewed the draft and provided feedback. All authors contributed to the article and approved the submitted version.

## Funding

The authors acknowledge the University of Florida—IFAS Seed Fund and USDA-NIFA Hatch Project #FLA-FTL-00565697 for financial support.

## Conflict of interest

The authors declare that the research was conducted in the absence of any commercial or financial relationships that could be construed as a potential conflict of interest.

## Publisher’s note

All claims expressed in this article are solely those of the authors and do not necessarily represent those of their affiliated organizations, or those of the publisher, the editors and the reviewers. Any product that may be evaluated in this article, or claim that may be made by its manufacturer, is not guaranteed or endorsed by the publisher.

## Footnotes

^1^https://www.sfwmd.gov/science-data/dbhydro
