# Supplementary material for: Spatiotemporal diversity and community structure of cyanobacteria and associated bacteria in the large shallow subtropical Lake Okeechobee (Florida, United States)
Source: Front Microbiol. 2023 Aug 30;14:1219261. doi: 10.3389/fmicb.2023.1219261 (PMC10499181; doi:10.3389/fmicb.2023.1219261)
Supplement: Supplementary file 2 [file Data_Sheet_2.DOCX]

Supplementary Material

Spatiotemporal diversity and community structure of cyanobacteria and associated bacteria in the large shallow subtropical Lake Okeechobee (Florida, USA)

Forrest W. Lefler , Maximiliano Barbosa , Paul V. Zimba, Ashley R. Smyth, David E. Berthold, H. Dail Laughinghouse IV^*^

*** Correspondence:** H. Dail Laughinghouse IV: [hlaughinghouse@ufl.edu](mailto:hlaughinghouse@ufl.edu)

**Supplementary Data 1**: Sampling location and limnological data collected during the course of this study.

**Supplementary Figure 1**: Time series plots of the total nitrogen total phosphorus ratio (TN:TP) at South Florida Water Management District (SFWMD) sampling locations in close proximity to our sampling locations. Data acquired from DBhydro (<https://www.sfwmd.gov/science-data/dbhydro>). Horizontal bar at 23 represents phosphorus limitation, horizontal bar at 9 represents nitrogen limitation. Samples between both horizontal bars represents co-limitation. Vertical black bars represent sampling dates.

**Supplementary Figure 2**: Bar plot of the average relative abundance of the most abundant bacterial phyla (A), cyanobacterial orders (B), and cyanobacterial families (C).

**Supplementary Figure 3**: Phylogenetic tree of cyanobacterial ASV’s within the Aphanizomenonaceae.

**Supplementary Figure 4**: Phylogenetic tree of cyanobacterial ASV’s within the Microcystaceae.

**Supplementary Figure 5**: Phylogenetic tree of cyanobacterial ASV’s within the Synechococcales.

**Supplementary Figure 6**: Phylogenetic tree of cyanobacterial ASV’s not classified to the genus level.


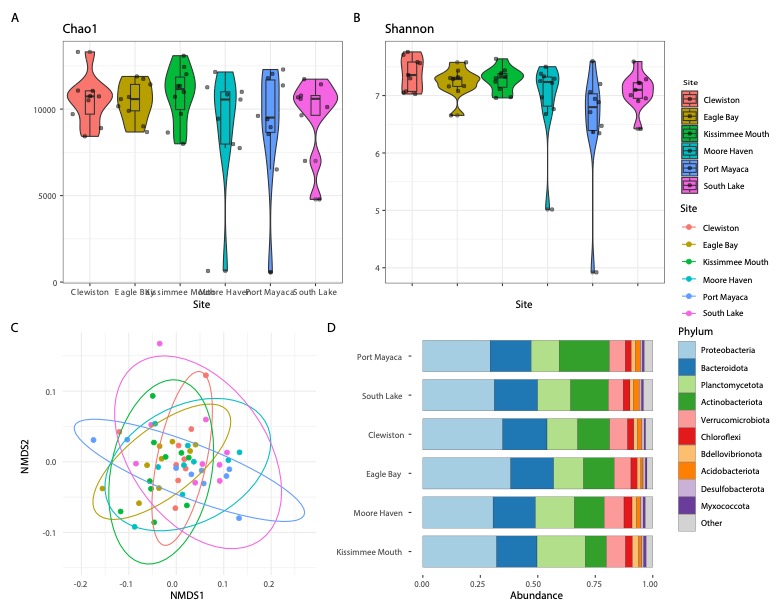


**Supplementary Figure 7**: Diversity measures of the bacterial communities at the different sampling locations. Chao1 index (A) and Shannon index (B) between each sampling location. Non-metric Multidimensional Scaling (NMDS) ordination within two dimensions of the cyanobacterial communities based on generalized-Unifrac distances between sampling locations, colors represent (C). Bar plot of the average relative abundance of the most abundant bacterial phyla at each location (D).

**Supplementary Figure 8**: Bar plot of the average relative abundance of the most abundant bacterial phyla during between season (A) and nutrient limitation (B). Non-metric Multidimensional Scaling (NMDS) ordination within two dimensions of the bacterial communities based on generalized-Unifrac distances between seasons, colors represent seasons (C). Bar plot of the average relative abundance of the most abundant bacterial phyla during each season (D).


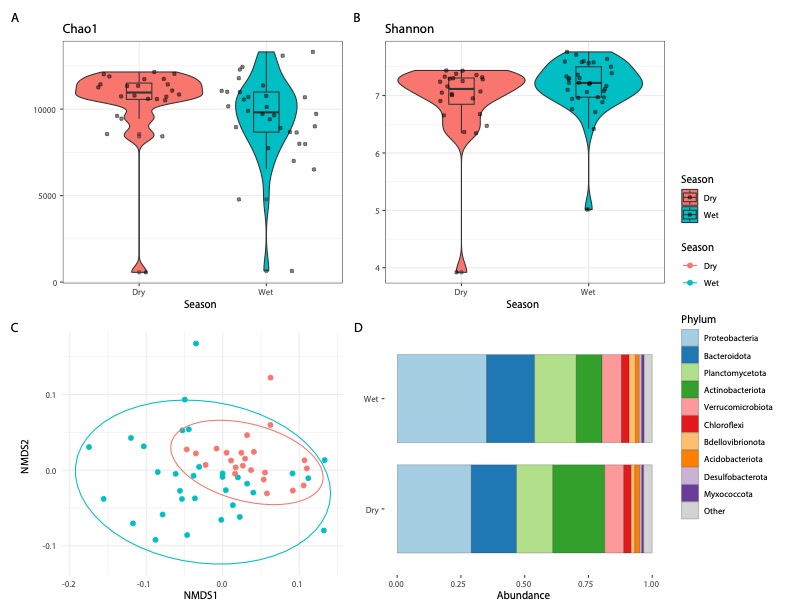


**Supplementary Figure 9**: Diversity measures of the cyanobacterial communities between wet and dry season. Chao1 index (A) and Shannon index (B) between each season. Non-metric Multidimensional Scaling (NMDS) ordination within two dimensions of the cyanobacterial communities based on generalized-Unifrac distances between sampling locations, colors represent season (C). Bar plot of the average relative abundance of the most abundant, non-Prochlorococcacean, bacterial phyla during each season (D).


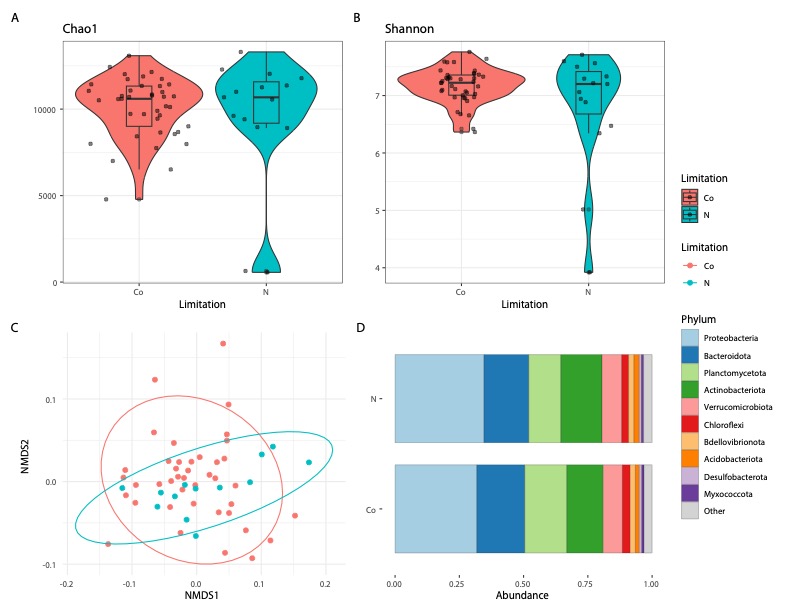


**Supplementary Figure 10**: Diversity measures of the bacterial communities between nitrogen and co-nutrient limitation. Chao1 index (A) and Shannon index (B) between nutrient limitations. Non-metric Multidimensional Scaling (NMDS) ordination within two dimensions of the bacterial communities based on generalized-Unifrac distances between nutrient limitations, colors represent limitation (C). Bar plot of the average relative abundance of the most abundant bacterial phyla during nutrient limitations (D).

**Supplementary Figure 11**: Effects of water temperature (A,D), Photic depth (B,E), and lake depth (C,F) on *Cyanobium* (A–C) and *Vulcanococcus* (D–F) as identified with generalized additive models (GAMs). Shaded areas indicate 95% confidence intervals, shapes indicate limitation, and colors indicate season.


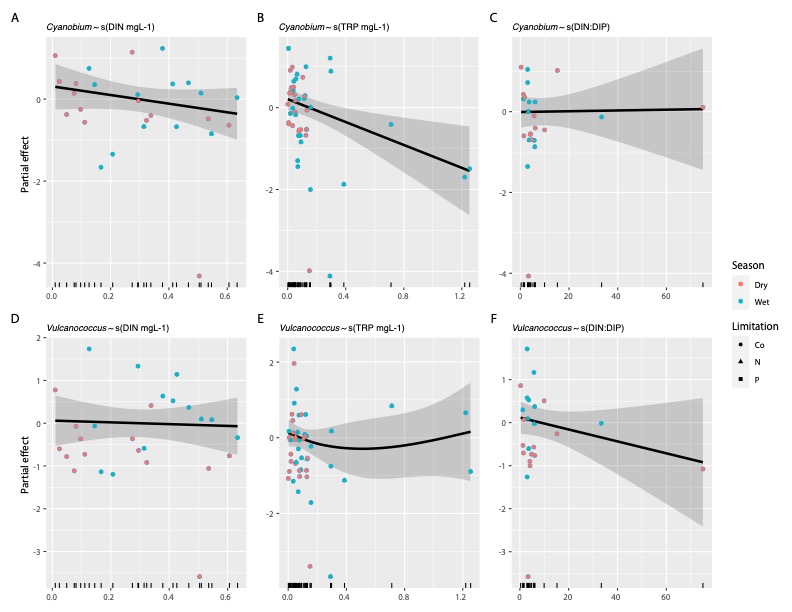


**Supplementary Figure 12**: Effects of dissolved inorganic nitrogen (A,D), total reactive phosphorous (B,E), and DIN:DIP (C,F) on *Cyanobium* (A–C) and *Vulcanococcus* (D–F) as identified with generalized additive models (GAMs). Shaded areas indicate 95% confidence intervals, shapes indicate limitation, and colors indicate season.

**Supplementary Figure 13**: Spearman correlation analyses between cyanotoxins and cyanobacterial genera.

**Supplementary Table 1**: Indicator taxa for each season.

| Taxon | Stat | p-value |
| --- | --- | --- |
|  | Dry Season | |
| Microcystaceae Cluster 2 | 0.470 | 0.0003 |
| *Aphanizomenon* | 0.462 | 0.0007 |
| *Woronichinia* | 0.444 | 0.0013 |
| *Regnicoccus* | 0.441 | 0.0012 |
| *Lacustricoccus* | 0.439 | 0.0006 |
| Prochlorococcaceae*_XX* | 0.421 | 0.0014 |
| *Raphidiopsis* | 0.383 | 0.0046 |
| *Synechocystis* | 0.380 | 0.0058 |
| *Metis* | 0.345 | 0.0128 |
| *Pegethrix* | 0.316 | 0.0235 |
| *Cuspidothrix* | 0.312 | 0.0216 |
| Leptolyngbyaceae Cluster 1 | 0.303 | 0.0285 |
| *Nodosilinea* | 0.276 | 0.0447 |
|  | Wet Season | |
| Synechococcaceae Cluster 1 | 0.664 | 0.0001 |
| *Apatinema* | 0.365 | 0.0062 |
| *Neocylindrospermum* | 0.301 | 0.0228 |

**Supplementary Table 2**: Cyanotoxins observed during this study.

| Site | Outing | MC-LR ppb | MC-RR ppb | Nodularin 811 ppb | Anatoxin-a ppb | Season |
| --- | --- | --- | --- | --- | --- | --- |
| Clewiston | 1 | 0 | 0 | 0 | 0 | Wet |
| Moore Haven | 1 | 0.07186697 | 0 | 0 | 0.48055224 | Wet |
| Eagle Bay | 1 | 0.3860031 | 0 | 1.1127493 | 0 | Wet |
| Clewiston | 3 | 0.15123439 | 0 | 0 | 0 | Wet |
| Kissimmee Mouth | 3 | 0 | 0 | 0.75171094 | 0 | Wet |
| Clewiston | 5 | 0.05882699 | 0 | 0 | 0 | Dry |
| Clewiston | 8 | 0.6 | 0.6 | 0 | 0 | Wet |
| Port Mayaca | 8 | 0.6 | 0 | 0 | 0 | Wet |
| Moore Haven | 8 | 0.6 | 0 | 0 | 0 | Wet |
| Clewiston | 9 | 0 | 0 | 0 | 0 | Wet |
| Clewiston | 6 | 0.12245818 | 0 | 0.67667188 | 0 | Dry |
| Eagle Bay | 6 | 0.31060651 | 0 | 1.12338534 | 0 | Dry |
| Clewiston | 7 | 0.19143283 | 0 | 0 | 0 | Dry |
| Port Mayaca | 7 | 0 | 0 | 0.25658517 | 0 | Dry |
| Eagle Bay | 7 | 0 | 0 | 0.84328562 | 0 | Dry |
| Kissimmee Mouth | 7 | 0.04421829 | 0 | 0.15293885 | 0 | Dry |
